# Total Polyphenol Contents and Mineral Profiles in Commercial Wellness Herbal Infusions: Evaluation of the Differences between Two Preparation Methods

**DOI:** 10.3390/foods13132145

**Published:** 2024-07-05

**Authors:** Vincenzo Lo Turco, Vincenzo Nava, Angela Giorgia Potortì, Benedetta Sgrò, Maria Aurora Arrigo, Giuseppa Di Bella

**Affiliations:** Department of Biomedical and Dental Sciences and of Morphological and Functional Images (BIOMORF), University of Messina, 98168 Messina, Italy; vloturco@unime.it (V.L.T.); vnava@unime.it (V.N.); benedetta.sgro@studenti.unime.it (B.S.); arrigoaurora@gmail.com (M.A.A.); gdibella@unime.it (G.D.B.)

**Keywords:** wellness herbal infusions, infusions preparation methods, polyphenols, mineral elements, transfer rates, health benefit assessments, health risk assessments

## Abstract

The popularity of the consumption of wellness herbal teas is due to the many health-promoting properties they seem to possess. Modern preparation methods using coffee machines are also popular today. Therefore, the purpose of this research was to evaluate differences in infusions obtained by the traditional method using filters and by espresso coffee machines using pods. In this regard, different herbal materials were selected and purchased in two different types of herbal containers, and the corresponding infusions were analyzed for the contents of total polyphenols and mineral elements. Results showed that filter infusions had higher polyphenol and mineral contents than pod infusions, excluding Cd and Pb. For each of the plant materials used, differences due to the method of infusion preparation are highlighted. From a qualitative point of view, both methods of infusion preparation are valid, but the filter infusion method allows a higher transfer of minerals and polyphenols into the infusion, improving quality. The analyzed infusions can be safely consumed with respect to As, Cd, Pb, and Hg contents. Good amounts of polyphenols and Mn can be obtained by drinking a cup of any of the infusions analyzed, especially the mate infusion obtained by the filter technique, with amounts of 429 mg for polyphenols and 69.27% of the RDA for manganese.

## 1. Introduction

Due to an increased awareness of the importance of good health, consumers have a particular interest in the eating habits of a healthy lifestyle and search for “healthy” foods and drinks, tisanes included. 

The tisane, also called an herbal infusion or herbal tea, is the infusion of a constituent of fresh or dried plant materials different from *Camellia sinensis* [1], from which true tea is obtained. The herbal infusions are made by the extractions of leaves, flowers, roots, or herbs of different botanical species in boiled water over time, through traditional infusion or using a suitable coffee machine. The most common herbal material used is chamomile, but fennel, licorice, and mint are also popular [2]. Exploiting water’s solvent capacity, organic substances with health effects present in the plants pass to the water, giving unique flavors, aromas, and possible medicinal properties to the beverage [3]. Generally, the tisanes are prepared immediately before use and consumed hot or cold within 12 h after infusion. Commercial tisanes are available in a variety of forms, including whole dried plant parts or bulk dried powders, in tea bags functioning as classic filters, or in pods for coffee machines.

The potential health benefits of herbal infusions have been studied since the 4th century BC, when Hippocrates prescribed an extract of willow bark as an analgesic and antipyretic, because it is rich in salicylates [4]. In fact, herbal infusions have long been used in traditional Eastern medicine for the presence of different phenolic compounds that protect the human body from free radicals’ damaging effects and different diseases such as cancer, cardiovascular diseases, and metabolic diseases such as diabetes [5,6,7].

Antioxidants are able to stabilize or deactivate free radicals before they attack cells and biological targets. This makes them essential to maintaining optimal cellular and systemic health and wellness [8,9,10]. 

Over the past few years, due to their sensory characteristics, low cost, low sugar content, and because they are free of theine and caffeine and a source of bioactive compounds, herbal infusions have become popular worldwide [11]. Tisanes are consumed as tea and coffee substitutes, to encourage water intake and to treat specific health disorders. For example, chamomile is often used for its calming properties [12], while fennel is indicated for intestinal disorders [13]. In addition, when consumed as part of a balanced diet, herbal infusions can improve antioxidant status, reducing oxidative stress and the incidence of related diseases [6].

In this regard, the World Health Organization planned the use of herbal medicines in the 2014–2023 strategy with the aim of keeping populations healthy through alternatives to medicine [14]. Actually, about 70–80% of the world’s population used alternative medicine, especially herbal infusions, as their first home remedy for health problems [15]. In Italy, the consumption of herbal infusions has increased significantly in the last three years. In fact, around 40% of Italians drink herbal infusions, mainly chamomile [16], and the market is worth EUR 200 million [17]. Consequently, there is a need to investigate the safety and quality of herbal preparations for brewed products.

During the infusion process, in addition to the organic bioactive substances, the mineral elements and different contaminants present in the herbs are also transferred to the water. So, the herbal infusions contain various mineral elements such as Na, Mg, K, and Ca on the order of mg/L and Fe, Mn, Cu, Al, As, Ba, Cd, Co, Cu, Cr, Ni, Pb, Se, V, and Zn on the order of µg/L [18,19]. The consumption of herbal tea may contribute to the dietary requirements of several essential elements. In fact, some mineral elements, such as potassium, calcium, sodium, magnesium, iron, and manganese, among others, play important roles in the human body [20], and their deficiency or imbalance could cause physiological disorders. For example, calcium is an integral part of the skeleton, and a deficiency can cause osteoporosis and rickets; iron is the building block of hemoglobin, so a deficiency causes decreased hemoglobin synthesis, resulting in anemia; and imbalances of magnesium and potassium are associated with changes in blood pressure and heart rhythm, or chronic fatigue [21]. In contrast, other minerals like copper, zinc, and nickel become toxic only in high concentrations, while mercury, lead, cadmium, and arsenic are harmful, even in small quantities [22]. According to the IARC (International Agency for Research on Cancer), Cd, Cr (VI), Ni, and As are classified as carcinogenic to humans (group 1); inorganic Pb as probably carcinogenic (group 2A); and metallic Ni, organic Pb, and MeHg as possibly carcinogenic (group 2B); and Cr (III), Hg, and inorganic Pb cannot be classified as carcinogenic to humans (group 3) [23]. 

In this regard, the European Commission Regulation (EC) No. 915/2023, repealing Regulation (EC) No. 1881/2006, established maximum levels for Hg, Pb, Cd, As, and Sn in different foods and beverages, but not in tea or herbal infusions [24]. 

The presence of mineral elements in herbal infusions depends on the environment (soil, water, and air), the agriculture practice, including the use of pesticides and fertilizers (i.e., lead arsenate), the production process, and the capacity of the plant to absorb and accumulate some elements [22,25]. As a consequence, it is desirable that herbs used for health purposes are grown in areas free from contamination [26].

In view of the difficulty of ensuring a non-contaminated area and the rapidly growing market and consumption, a nutritional and toxicological characterization of the herbal infusions available in Italy would provide important data to assess their safety.

The aims of this study were to investigate the contents of total polyphenols and mineral elements in commercial wellness herbal infusions from different herbal materials and to evaluate the differences between two different infusion methods (traditional infusion and using a coffee machine). In this regard, the spectrophotometric method was used to evaluate the total polyphenolic content, while the mineral elements were determined by ICP-MS and Hg by DMA-80. Thus, the concentrations of total polyphenols and mineral elements were expressed for the herbal material and the two types of infusions. Moreover, the transfer rate from the herbal materials to the infusions of these elements (total polyphenols and minerals) and their contributions to the reference values with respect to the consumption of one cup of each infusion were evaluated.

## 2. Materials and Methods

### 2.1. Chemicals and Reagents

Throughout the experiment, ultrapure water at a resistivity of 10 MΩ cm (J.T. Baker, Milan, Italy) was used. Solvents and reagents of ultrapure grade, such as acetonitrile, HNO_3_ (65% *v*/*v*), and H_2_O_2_ (30% *v*/*v*) were purchased from Merck (Darmstadt, Germany). Reference standard solutions of gallic acid and the Folin–Ciocalteu reagent were obtained from Sigma-Aldrich (Darmstadt, Germany). Standard solutions of inorganic elements, such as Mg, Ca, Mn, Fe, Co, Cu, Zn, Cd, Ba, Cr, Li, B, Na, Al, Ni, Mo, K, Pb, As, and Se at a concentration of 1000 mg/L in 2% HNO_3_ (Fluka, Milan, Italy) were used for the preparation of multielement stock standard solutions at the concentration of 100 mg/L for each element. Hg solution (1000 mg/L in 3% HCl) was obtained from Merck (Darmstadt, Germany). The online internal standard solutions of ^45^Sc, ^73^Ge, ^115^In, and ^209^Bi (1000 mg/L in 2% HNO_3_) and the internal standard solution of Re (1000 mg/L in 2% HNO_3_) were obtained from Fluka (Milan, Italy) and were used to correct instrumental drift and matrix deviation, and to check the sample digestion and the volumetric changes, respectively. Standard Reference Material Trace Elements in Spinach Leaves (SRM, NIST 1570a) was obtained from the National Institute of Standards and Technology (Gaithersburg, MD, USA).

For element analysis, all laboratory equipment was washed with 5% HNO_3_ before use to avoid undesirable metal contamination.

### 2.2. Samples

During the month of September 2023, seven types of herbal materials for infusion were selected and purchased in 2 different types of herbal containers, classic filters and pods for espresso coffee machines. The filters were made of filter paper, flat and rectangular in shape and filled with herbal materials. The pods consisted of herbal material sealed between two sheets of filter paper. Three different brands were sampled for each type to ensure homogeneity. Samples in filters (*n* = 21) were organic and were obtained from herbal shops located in Messina (Sicily, Italy), while samples in pods (*n* = 21) were obtained from an online shop. The seven herbal types were chamomile (*Matricaria camomilla*), fennel (*Foeniculum vulgare*), licorice (*Glycyrrhiza glabra*), mate (*Ilex paraguensis*), mint (*Mentha piperita*), moringa (*Moringa oleifera*), and red rooibos (*Aspalatus linearis*). The parts of the plants that made up the samples were flowers for chamomile; seeds for fennel; roots for licorice; and leaves for mate, mint, moringa, and red rooibos. Specific criteria were considered for the product selection: (i) the herbal infusions consist of one main ingredient; and (ii) the pods and filters must not have staples. All samples were kept in their original packages at room temperature in a dry and dark place until the analysis and opened just before analysis to avoid oxidative damage. The geographical origins of the herbs used for this study, the season in which they were harvested, and the conditions under which the herbs were dried were unknown because label data were lacking.

### 2.3. Preparation of Herbal Infusions

Each herbal infusion was prepared in triplicate according to the manufacturer’s instructions. For herbal infusion preparation using the traditional method, the filters were filled with 250 mL of distilled water at 100 °C in glass bottles for a brewing time of 5 min. The “Didiesse Frog” coffee machine, previously purged with 500 mL of distilled water, was used for the preparation of herbal infusions using herbal pods. The pods were placed in the machine, and after about 30 s, 250 mL of the herbal infusion was collected in a glass bottle. The herbal infusions were filtered and collected in 50 mL graduated tubes before the total polyphenols and inorganic elements determination. 

### 2.4. Determination of Total Polyphenols

The total polyphenol contents (TPCs) were determined following the method described by AlHafez et al. [27], with modifications. For the extraction of polyphenols from the herbal material, 1 g of each sample was added to 50 mL of acetonitrile–water (50% *v*/*v*) in a flask and incubated in an agitated thermostatic water bath at 75 °C for 2 h. The extract was filtered, the acetonitrile was removed by a rotary evaporator at 40 °C, and the volume of the remaining aqueous solution was completed to 25 mL. Then, 1 mL of each herb’s aqueous extracts was diluted to 100 mL with distilled water, and the determination of TPC was carried out by the spectrophotometric method using the Folin–Ciocalteu reagent. Briefly, 1 mL of each diluted sample was mixed with 4.8 mL of distilled water, 4 mL of Na_2_CO_3_ (2%), and 200 μL of Folin–Ciocalteu reagent. The samples were incubated in the dark at room temperature for 60 min; then, the absorbance was measured at a wavelength of 750 nm with a UV–VIS spectrophotometer (UV-2401 PC, Shimadzu, Milan, Italy).

For herbal infusions, each extract of 1 mL was diluted to only 50 mL using distilled water, and then 1 mL of each diluted sample was treated as described above. 

For total polyphenols quantification, a six-point calibration curve was constructed using gallic acid (slope = 0.0027; R^2^ = 0.9981), and the TPC was expressed as the gallic acid equivalent. 

### 2.5. Determination of Mineral Elements

#### 2.5.1. Samples Preparation

Of each herbal material, 0.5 g was added to 7 mL of HNO_3_, 1 mL of H_2_O_2_, and 1 mL of the internal standard Re (0.5 mg/L) in PTFE (polytetrafluoroethylene) vessels. The mineralization was carried out through a microwave ETHOS 1 digestion system (Milestone, Bergamo, Italy) with the following instrumental parameters: 10 min from 0 °C to 200 °C, then 10 min held at 200 °C, with a microwave power in the range of 1000–1100 W, and 20 min for cooling down [28]. Then, the digested samples were conveniently diluted with ultrapure water to 25 mL, filtered through 0.45 µm PTFE filters, and analyzed by ICP-MS.

Each herbal infusion was acidified with 2% nitric acid (HNO_3_), filtered through 0.45 µm PTFE filters, and analyzed by ICP-MS.

#### 2.5.2. ICP-MS Analysis

The determination of mineral elements was carried out by a quadrupole ICP-MS, iCAP-Q (Thermo Scientific, Waltham, MA, USA) equipped with an ASX-520 autosampler (Cetac Technologies Inc., Omaha, NE, USA).

The ICP-MS operating conditions are summarized in Table S1 in the Supplementary Materials [29].

For mineral quantification, five-point calibration curves were built up for each analyte with internal standard normalization. Multielement standard solutions, certified samples, and analytical blanks were used, and all the samples were analyzed three times with the same conditions.

#### 2.5.3. Determination of Hg

The Hg content was evaluated through a DMA-80 Direct Mercury Analyzer (Milestone S.r.l., Milan, Italy). After instrument cleaning with 3% HCl solution, samples were analyzed directly without pretreatment. The herbal materials (0.1 g) were placed in nickel boats, while the infusions (100 µL) were placed in quartz boats. The samples, before being placed into the instrument, were first dried at 250 °C for 60 s and then thermally decomposed into an oxygen stream at 750 °C for 150 s. The Hg’s quantitative determination was performed by measuring the absorbance at 253.7 nm, using a five-point calibration curve. Standard solutions, certified samples, and analytical blanks were used, and all the samples were analyzed in three replicates with the same conditions [30].

### 2.6. Analytical Performances

The EURACHEM criteria were followed for the validation procedures [31]. The linearity, sensibility, accuracy, repeatability, and intermediate precision of the analytical method were assessed. 

The linearity of the calibration curves was evaluated by the respective correlation coefficients (R^2^ values). Limits of detection (LODs) and limits of quantification (LOQs) were 3.3 σ/S and 10 σ/S, respectively, where σ represents the standard deviation of the response of ten blanks, and S is the slope of the calibration curve. For the analysis of herbal materials, the reference material NIST 1570a (spinach leaves) was analyzed to check the accuracy. The mineral elements not present in the certified matrix were added at a concentration of 1 ppm each. In the case of herbal infusions, the recovery was checked by the analysis of a sample (previously analyzed) to which known amounts of elements were added. The method’s precision was studied for repeatability and intermediate precision. Repeatability and intermediate precision were quantified based on the relative standard deviation (RSD %) of measurements made for the same batch and on different days. 

Table S2 in the Supplementary Materials summarizes the results of the method’s validation. The performance parameters of the method are satisfactory for the elements considered. The calibration lines were all linear, with R^2^ values greater than 0.9976. LOQ values were estimated to be between 0.002 and 1.962 µg/kg. The results for accuracy for herbal materials were between 91.97 and 102.50%. The results for recovery for herbal infusions were between 93.18 and 102.19%. The results for the repeatability and intermediate precision of the applied method were lower than or equal to 5.11% and 6.89%, respectively.

### 2.7. Health Benefits and Risk Assessment

The dietary intake of polyphenols and mineral elements through the consumption of herbal infusions was estimated.

The estimated daily intake (*EDI*) was calculated for essential elements by the following equation:

*EDI* (mg/day or μg/day) = *C* (mg/L or μg/L) *× I* (L/day) (1)

where *C* is the concentration of each element in the analyzed samples and *I* is the intake for one cup of herbal infusion (0.25 L). For toxic and potentially toxic elements, we used the following equation:

*EDI* (mg/kg_bw_/day or μg/kg_bw/_day) = [*C* (mg/L or μg/L) × *I* (L/day)]/kg_bw_ (2)

where kg_bw_ is a normal adult’s bodyweight (70 kg).

The *EDI* for total polyphenols was also estimated.

The *EDI*s for mineral elements were compared with the following reference values: AI, Adequate Intake, for sodium [32]; RDA, Recommended Dietary Allowance, for the other essential elements [33] and lithium [34]; TDI, Tolerable Daily Intake, for barium [35] and nickel [36]; TWI, Tolerable Weekly Intake, for aluminum [37] and cadmium [38]; UL, Tolerable Upper Intake Level, for boron [39]; and BMDL01, Benchmark Dose Lower Confidence Limit 01, for arsenic [40] and lead [41]. The results were expressed as percentages of the reference values.

### 2.8. Statistical Analysis

Statistical analyses were conducted on three datasets using the SPSS 13.0 software package for Windows (SPSS Inc., Chicago, IL, USA). For the first one, the starting multivariate matrix was constituted of 42 cases (samples of herbal materials for the infusions under analysis) and 22 variables (the total polyphenol contents and the concentrations of elements determined in the analyzed samples). The dataset was divided into seven groups according to the type of herbal material used in the infusion. The second dataset concerned the infusions obtained from the herbal materials, where the matrix with 42 cases (infusion samples analyzed) and 21 variables (total polyphenol contents and concentrations of elements determined in the analyzed samples) was also divided into seven groups according to the type of herbal material from which the infusions were obtained, and then it was further divided into two groups according to the infusion preparation (traditional method from filters and by espresso coffee machine from pods). Mercury was not included in the latter dataset because it was always < LOQ. If concentrations of certain elements were below the LOQ in only a few samples, they were replaced by the LOD/2 value [42]. For the third dataset, related to transfer rate values, the initial multivariate matrix consisted of 42 cases (analyzed samples) and 22 variables (transfer rate values for total polyphenols and element concentrations).

The Kruskal–Wallis test was used to compare the concentrations of polyphenols and mineral elements in samples from different botanical origins with each other, while the Mann–Whitney U test was used to assess the differences between the two methods of infusion preparation. Differences were considered statistically significant at *p* < 0.05. 

To visualize the arrangement of the samples of the different herbal materials for the infusions analyzed (chamomile flowers, fennel seeds, licorice roots, mate leaves, mint leaves, moringa leaves, and red rooibos leaves) in an n-dimensional space where most of the information is retained, a Factor Analysis by Principal Components extraction was performed. First, all independent variables, indicated in the respective starting multivariate matrix, were entered together, and were normalized [43], and then the data were checked for suitability for factor analysis.

## 3. Results and Discussion

### 3.1. Total Polyphenols

The total polyphenol contents of the herbal materials and their respective infusions are reported in Table 1.

The average concentrations of total polyphenols in the herbal materials varied from 158.64 ± 10.65 mg GAE/g to 15.74 ± 1.85 mg GAE/g, while in the herbal infusions they varied from 171.60 ± 14.97 mg GAE/g to 13.45 ± 1.01 mg GAE/g in the infusions obtained with the traditional method and from 69.03 ± 5.44 mg GAE/g to 15.37 ± 1.63 mg GAE/g in the infusions obtained through the coffee machine.

The results showed that for the herbal materials, mate and red rooibos leaves had the significantly highest levels of TPCs, while fennel seeds had the lowest levels. The same was true for herbal infusions, except for red rooibos, whose TCP value was not significantly different from that of mint and moringa.

In general, the polyphenol contents of the herbal materials and herbal infusions were similar to those found in the literature data. De Meja and colleagues [44] found a content of polyphenols in dried mate leaves that varied from 90.4 ± 9.9 to 176.1 ± 15.6 mg GAE/g. The TPC values of our sample fall into the same range. 

Ilyas et al. [45] evaluated the TPC in moringa leaves. As a result, they found that the moringa leaves showed a value of 9535.3 ± 57.74 mg GAE/100g, which is an intermediate value between the TPCs of moringa leaves that we found in infusions produced through pods and filters (81.79 ± 7.43 and 111.11 ± 11.23 mg GAE/g, respectively). 

Damiani et al. [46] evaluated the TPC of red rooibos infusions produced in 200 mL of hot water infused for 5 min, obtaining a result lower than that of our infusion produced through the classic filter technique but slightly higher than that of herbal tea made using the coffee machine. 

Rusaczonek et al. [47] evaluated the TPCs in mint and chamomile herbal infusions; the chamomile infusion showed an average of 44 ± 7.9 mg GAE/g and the mint infusion showed a value of 90 ± 0.3 to 201 ± 0.3 mg GAE/g, values that are similar to our results.

Concerning the infusions made with pods using the coffee machine, the highest TR was obtained for chamomile (70.99 ± 5.27%), while the lowest TR was obtained for mate (48.73 ± 1.31%). For infusions made with traditional filters, the highest TR was obtained for mate (97.13 ± 1.93%), while the lowest TR was obtained for licorice (43.73 ± 5.46%). As suggested by Theuma & Attard [48], this could be due to whether the compound is soluble in the infusion or whether it is strongly bound to the matrix due to the formation of complexes with other metabolites. It is also possible that it may be bound to the filter or pod materials.

Although different TRs were observed depending on the plant material, on average, the infusions obtained with filters had significantly higher TR values than those obtained with pods. The reason is probably related to the temperature of the water and the time taken to brew the herbal infusion (higher for traditional preparation). 

### 3.2. Elemental Contents in Herbal Materials

Table 2 shows the levels of elements in the herbal materials studied. 

In the herbal materials studied, the elements of interest were measured at various concentrations. The wide variation in element concentrations could be attributed to the varying abilities of plants to uptake and translocate the elements. Metal uptake by plants depends on several factors. These include the plant species and its growth stage, the soil characteristics, the climatic conditions and the geo-environmental properties (such as pH, oxidation–reduction potential), the anthropogenic activities (pollution, industrial sites), etc. [15,49,50]. 

The main elements detected were K, Mg, and Ca. The highest K contents were in fennel seeds (18.74 ± 5.53 g/kg) and mint leaves (18.61 ± 5.77 g/kg), while the lowest was in red rooibos leaves (3.30 ± 1.70 g/kg). The Mg content was highest in moringa leaves (10.81 ± 0.35 g/kg), and the Ca content was highest in mint leaves (12.67 ± 0.67 g/kg). The magnesium content was lower in red rooibos leaves (2.19 ± 0.25 g/kg), while the Ca contents were lower in red rooibos leaves (1.62 ± 0.28 g/kg), licorice roots (2.19 ± 0.13 g/kg), and chamomile flowers (2.22 ± 0.14 g/kg). Chamomile flowers, fennel seeds, licorice roots, and mate leaves showed a proximity between Ca and Mg concentrations, while in mint and moringa leaves, the results highlight the proximity between Ca and K concentrations.

The data analysis revealed that the Na content in the red rooibos samples was 2739.90 ± 248.04 mg/kg, which is very different from the other herbal materials (from 100.52 ± 7.23 mg/kg in mint leaves to 810.35 ± 57.23 mg/kg in fennel seeds). 

Concerning essential trace elements, the most abundant were Fe, Mn, Zn, and Cu. In addition to high K and Ca contents, mint leaves showed the highest concentrations of Fe (853.17 ± 46.83 mg/kg) and Cu (15.18 ± 2.77 mg/kg). Mate and red rooibos leaves were found to have very low Fe contents (9.85 ± 2.70 and 9.05 ± 1.52 mg/kg, respectively). Mate leaves had the highest content of Mn (756.57 ± 146.62 mg/kg). The Zn concentrations were high in mint (38.72 ± 3.70 mg/kg) and mate (35.41 ± 0.92 mg/kg) leaves. The concentrations of Cr, Mo, Co, and Se were less than 1.50 mg/kg in all samples. In the case of Cr, the highest levels were found in leaves of mint (1.17 ± 0.15 mg/kg), and then in flowers of chamomile (1.06 ± 0.31 mg/kg); the highest Mo contents were in mint leaves (0.98 ± 0.32 mg/kg) and chamomile flowers (0.87 ± 0.28 mg/kg), while the lowest was in mate leaves (0.03 ± 0.02 mg/kg); the Co mean concentration in leaves of mate (0.35 ± 0.05 mg/kg) was significantly higher than in the other samples; the Se contents were higher in fennel seeds (0.20 ± 0.06 mg/kg), chamomile flowers (0.18 ± 0.058 mg/kg), and red rooibos leaves (0.16 ± 0.04 mg/kg).

Among the herbal materials studied, mint was found to be the most abundant in minerals and essential trace elements (especially K, Ca, Fe, Zn, Cu, Cr, and Mo), while red rooibos was the least abundant in most of the same elements, but the most abundant in Na.

The concentrations of Al found in the herbal materials studied were very variable: from 6.10 ± 1.38 mg/kg (in red rooibos leaves) to 597.40 ± 10.19 mg/kg (in moringa leaves). The high values of Al were such that they could be compared to those of Na, Fe, and Mn. In addition to their high Al content, moringa leaves showed the highest Li concentration (0.426 ± 0.120 mg/kg). This value differs greatly from other plant materials (from <LOQ in licorice roots and mint leaves to 0.070 ± 0.014 mg/kg in mate leaves).

The highest levels of B (53.65 ± 3.91 mg/kg), Ba (27.60 ± 7.44 mg/kg), and Ni (3.22 ± 0.63 mg/kg) were found in the leaves of the mate.

Very low levels of contamination with toxic elements were found in the samples. The highest As concentration, 0.132 ± 0.059 mg/kg, was found in red rooibos leaves. Pb contents ranged between 0.026 ± 0.019 mg/kg (in mate leaves) and 0.071 ± 0.043 mg/kg (in red rooibos leaves). There were no significant differences among the plant materials (*p* > 0.05) in this case. Among the analyzed groups of herbal materials, the most contaminated with Cd turned out to be the leaves of mate, where its concentration reached 0.223 ± 0.078 mg/kg on average. Meanwhile, the Cd contents were low in fennel seeds (0.006 ± 0.001 mg/kg), moringa leaves (0.012 ± 0.010 mg/kg), licorice roots (0.024 ± 0.020 mg/kg), and mint leaves (0.026 ± 0.026 mg/kg). The Hg contents of mate, mint and moringa leaves were very similar (between 0.006 ± 0.001 and 0.008 ± 0.002 mg/kg); there was no Hg in the other samples. The analyzed samples can be considered safe, considering that the permissible limits for Pb, Cd, and Hg are set at 5, 1, and 0.2 mg/kg, respectively, in the *European Pharmacopoeia* [51].

The concentrations of K, Mg, Ca, Na, Fe, Mn, Zn, Cu, Cr, and Co in chamomile and mint were largely consistent with those obtained by Derya Kara [52] in similar samples from Turkey. Malik et al. [49] reported the concentrations of Al, B, Cu, Fe, Mn, Zn Ca, K, and Mg in traditional plants, including mate, rooibos, and chamomile. Their results for mate were similar to those listed in the present study, with a few exceptions (Al, Ca, and Fe). On the contrary, their results for rooibos leaves differed from those from our samples in Al, B, Cu, Fe, and Mn concentrations. With regard to chamomile, the Al, B, Cu, Fe, Zn, Ca, and K contents they found were higher, while the Mn and Mg contents were lower than our results. In rooibos leaves from South Africa, as reported by Olivier et al. [20], the Ca, Mg, and K contents were similar to those found in the present study, while Fe, Mn, Zn, Cu, and Al contents were higher than those found in the present study. Mate leaves from South America were studied in the same paper: the contents of Mg, K, Cu, and Na were similar, while the other elements were higher than those determined in the present study. The concentrations of Pb in chamomile and mint were considerably lower than those found by Ababneh [53]. Salawu et al. [54] found lower amounts of Mg, Na, K, Zn, Fe, and Mn and higher amounts of Ca in moringa leaves and licorice roots than were found in the present study. Cd and Cu contents in mint, chamomile, and fennel, Zn and Mn contents in mint, and the Fe content in chamomile in our samples were in line with those reported by Sembratowicz & Rusinek-Prystupa [55].

### 3.3. Principal Component Analysis on Herbal Materials Data Set

Principal Component Analysis (PCA) was applied to the results to correlate the samples with their botanical group. PCA allows the highlighting and visualizing of the differences among the samples as well as the relationships between observations and variables. The data were suitable for factor analysis. All the variables were significantly correlated with at least one other variable. The KMO (Kaiser–Meyer–Olkin) value was 0.658; the approximate chi-square value was 1347.104, with a statistical significance at a *p*-level below 0.001; the determinant value was low (1.52 × 10^−18^); and 62% of the coefficients had values with a significance higher than 95%. The highest positive correlations were observed between Cu and Hg (0.945), Cu and As (0.913), and Cr and Ni (0.913), while only three negative correlations were observed, between Cd and Pb (−0.213), V and Hg (−0.178), and V and Pb (−0.104).

According to the Kaiser criterion, six principal components (PCs) were extracted with eigenvalues greater than one (6.105, 5.189, 3.572, 1.1956, 1.152, and 1.035). They cumulatively explained 86.40% of the total variance (27.748%, 23.585%, 16.236%, 8.893%, 5.237%, and 4.704%, respectively). Variables with low saturation in each factor were not identified. Communalities were always higher than 0.702. The first component is most positively correlated with Hg (0.855), Cu (0.825), Ca (0.818), and Fe (0.805), while Na (−0.688) is negatively correlated. Mn and, to a lesser extent, Cd, B, Ni, Ba, and TPC are correlated with the second component, all with positive values (0.893, 0.796, 0764, 0.744, 0.716, and 0.713, respectively). The dominant variables in the third component are Li (0.897), Al (0.777), Mg (0.661), and K (−0.556), while in the fourth component, the dominant variable is As (0.752); in the fifth, it is Cr (−0.550), and in the sixth, it is Pb (0.440).

**Figure 1:**
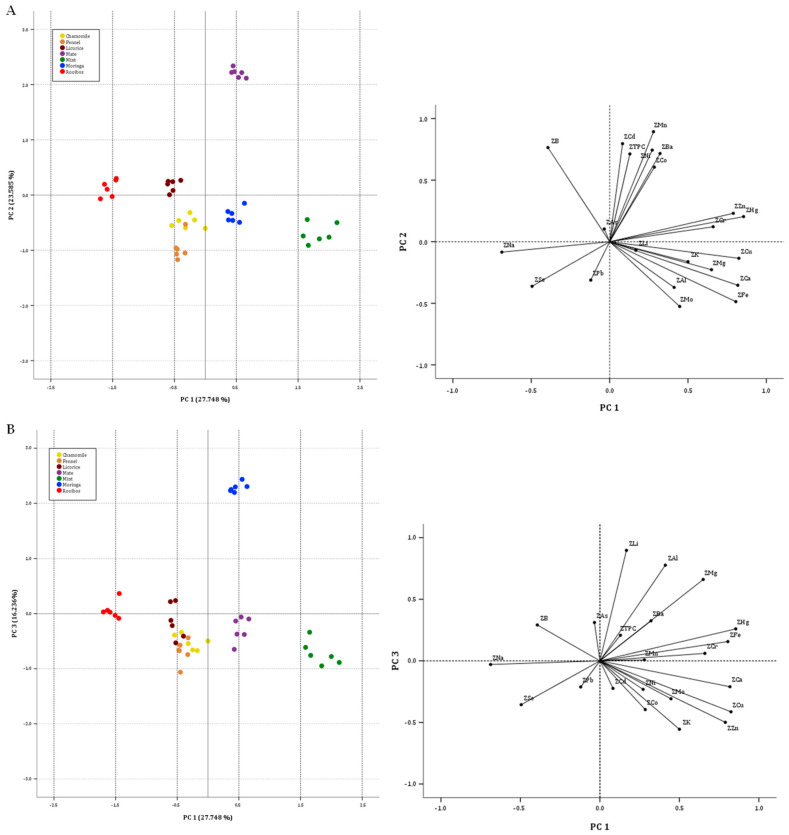
Two-dimensional score plots for the 42 herbal material samples categorized by botanical specie. ((**A**), PC2 against PC1 plot; (**B**), PC3 against PC1plot). Insert: loading plot of minerals in the spaces defined by PCs.

Figure 1A shows the plot of the PC1/PC2 scores. There is a slight tendency for the samples of the same category to be grouped together. The PC2 score is split between the mate, mint, and moringa samples and the rest of the plant materials. The first always showed positive PC1 values, while the rest showed negative PC1 values. The mate samples, on the other hand, were separated from the mint and moringa samples by PC1. They showed positive PC2 values and differed strongly in the content of total polyphenols, manganese, cobalt, boron, barium, cadmium, and nickel, with which a direct correlation can be observed. The mint, followed by the moringa samples (with negative PC2 values), had higher levels of iron, copper, calcium, zinc, and mercury and lower levels of sodium than the others. With respect to samples with negative PC1 scores, a good separation between the licorice and red rooibos group and the chamomile and fennel group can be observed. Licorice and red rooibos samples had positive PC2 values, while chamomile and fennel samples had negative PC2 values. The common characteristic of licorice and red rooibos samples was that they had the lowest copper contents; red rooibos was also strongly influenced by its very high sodium content. Chamomile and fennel samples had the lowest TPC values; they also had higher levels of selenium and low levels of manganese (along with red rooibos, which is shifted to the left due to its high sodium content). In addition, the plot in Figure 1B (PC1/PC3 scores) shows the moringa samples separating from the others on the PC2 axis due to significantly higher lithium, aluminum, and magnesium contents.

### 3.4. Elemental Contents in Herbal Infusions

The elemental contents of herbal infusions are listed in Table 3. The same variability in mineral profiles observed in the herbal materials was also maintained in the infusions. The contents of elements in the infusions also depend, in addition to the factors already mentioned for plant materials, on the part of the plant used (flowers, leaves, or roots) and on the infusion methods (temperature, time, amount of water, etc.) [15,49,50].

Overall, the results showed that the mineral content of filter infusions was higher than that of pod infusions.

Except for moringa, which had the highest Mg content, K was always the most abundant element. The K concentration varied from 6.69 ± 0.31 mg/L to 115.35 ± 3.23 mg/L; the Mg concentration varied from 5.13 ± 0.35 mg/L to 70.66 ± 1.63 mg/L; and the Ca concentration varied from 3.71 ± 0.41 mg/L to 71.96 ± 3.21 mg/L. The minimum values for each of these three elements were determined in red rooibos infusions obtained from pods, while the maximum values of K and Ca were determined in mint infusions obtained from filters, and the maximum value of Mg was determined in moringa infusions obtained from filters. 

The Na concentration was lower than 2.00 mg/L in all infusions obtained from chamomile, mate, mint, moringa, and licorice. Red rooibos infusions had the highest concentrations of Na (7.94 ± 0.19 mg/L and 16.60 ± 0.09 mg/L from pods and filters, respectively); this was followed by the values in fennel infusions (2.18 ± 0.13 mg/L and 5.55 ± 0.14 mg/L from pods and filters, respectively).

The highest concentrations of Fe were found in both mint infusions (5.37 ± 0.44 and 1.90 ± 0.23 mg/L in filter and pod teas, respectively), while the highest concentrations of Mn were found in both mate infusions (5.54 ± 0.30 and 2.10 ± 0.14 mg/L in filter and pod infusions, respectively). Mint and mate infusions also showed the highest contents of Zn (158.50 ± 20.81 and 149.83 ± 79.72 µg/L, respectively, averaged from filters and pods); and mint infusion also had the highest contents of Cu (54.27 ± 12.91 µg/L, averaged from filters and pods).

The highest Cr concentrations were found in infusions obtained from chamomile (3.27 ± 0.50 µg/L, averaged from filters and pods) and moringa (3.02 ± 0.83 µg/L, averaged from filters and pods), values which were significantly higher than those of the other plants. Mint and chamomile infusions had higher concentrations of Mo (averages from filters and pods: 4.55 ± 2.06 and 4.08 ± 2.12 µg/L, respectively) than other samples. Contents of Co were significantly higher in mint, mate, and licorice (averages from filters and pods: between 1.22 ± 0.53 µg/L and 1.55 ± 1.06 µg/L) than in chamomile, fennel, moringa, and rooibos infusions (averages from filters and pods: between 0. 52 ± 0.21 µg/L and 0.77 ± 0.35 µg/L), while Se contents were significantly higher in chamomile, fennel, and red rooibos infusions (averages from filters and pods: between 0.64 ± 0.30 µg/L and 0.83 ± 0.26 µg/L) than in licorice, mate, mint, and moringa infusions (averages from filters and pods: between 0.26 ± 0.15 µg/L and 0.38 ± 0.35 µg/L).

The contents of Al in mint infusions were 2.87 ± 0.17 and 1.15 ± 0.17 mg/L from filter and pod teas, respectively, while the highest Al contents of the other samples were 0.60 ± 0.12 and 0.49 ± 0.11 mg/L (from filters and pods, respectively). B in mint infusions was very low, and its amount was 9.17 ± 2.86 µg/L (averaged from filters and pods), while in mate infusions, it was considerably higher (217.00 ± 0.10 µg/L, averaged from filters and pods). In addition to having the highest B content, mate infusions also had the highest Ba (112.60 ± 55.23 µg/L, averaged from filters and pods) and Ni (11.48 ± 4.31 µg/L, averaged from filters and pods) contents. These values were significantly different from infusions made from other plant materials (averages from filters and pods: between 5.35 ± 2.92 µg/L and 72.25 ± 7.72 µg/L for Ba; between 2.17 ± 1.19 µg/L and 5.05 ± 2.99 µg/L for Ni). Li was below its limit of quantification in 43% of the infusions (all licorice and mint infusions obtained from both filters and pods, and all fennel and red rooibos infusions obtained from pods). Among the remaining infusions, the highest value was found in moringa infusions obtained from filters (1.044 ± 0.299 µg/L).

In the European Union, the permissible limits for As, Cd, Pb, and Hg in drinking water are 10, 5, 10, and 1 μg/L [56]. These limits were not exceeded in any of the samples analyzed, so they can be safely consumed. Regarding As, there was no significant difference found among the infusions obtained from different herbal materials: the concentrations (averaged from filters and pods) ranged from 0.060 ± 0.041 µg/L (in fennel infusions) to 0.373 ± 0.298 µg/L (in red rooibos infusions). Pb was below the limit of quantification in all mint infusions obtained from filters (7% of samples), Cd in 36% of the infusions (all fennel infusions obtained from both filters and pods, and licorice, mint, and moringa infusions obtained from filters), and Hg in 100% of the infusions. Fennel, licorice, mate, and moringa infusions had the lowest concentrations of Pb (0.064 ± 0.023, 0.052 ± 0.032, 0.040 ± 0.023, and 0.063 ± 0.041 µg/L, respectively), while chamomile, mint, and red rooibos infusions had values significantly higher (0.155 ± 0.058, 0.134 ± 0.155, and 0.172 ± 0.081 µg/L, respectively). The highest Cd concentrations were obtained from the infusions of mate leaves (0.507 ± 0.266 µg/L, averaged from filters and pods), with values significantly higher than those of the other herbal materials.

In the literature, several studies about mineral elemental contents in different herbal infusions are available, and the results highlighted a great variability among them. However, data on licorice infusions are rather limited. The concentrations of K, Na, Co, Cr, and Li in chamomile, of Cd, Co, and Pb in fennel, and of B, Cd, Cr, and Pd in mint infusions obtained in this study are similar to those obtained by Özcan et al. [19]. Concerning other mineral elements, the same authors found concentrations lower than our results, except for As in chamomile and fennel, Li in fennel and mint, Co and Al in mint, and Cr in fennel infusions, which showed higher concentrations. 

Puig et al. [57], in rooibos infusions, found higher K, Zn, Cu, and Ni, and similar Mg, Ca, Na, and Fe concentrations than those found in this work. 

Milani et al. [58] focused on essential and non-essential trace elements in mate infusions: the reported concentrations of Fe, Se, Ni, and As were consistent with our results, while the concentrations of other trace elements were lower than our results, except for Pb. 

Mate and chamomile infusions were also evaluated by de Oliveira et al. [59]. Mate infusions showed similar concentrations of Al and Cd, lower contamination of As, and higher contaminations of Cr and Pb than our results. The Cd content of the chamomile infusions was consistent with that found in our study, while the Al and Cr contents were lower, and the As and Pb contents were higher than our results. 

Moringa infusions analyzed by Ilyas et al. [45] were characterized by contents of Ca, Mg, and K lower than those of our samples, while Fe, Zn, Cu, Mn, Cr, Pb, and Cd were detected at higher concentrations than our samples. The Na contents were in line with our results.

### 3.5. Principal Component Analysis on Herbal Infusions Data Set

PCA was used to try to discriminate among infusion samples obtained by two types of infusion. The factorability of the correlation matrix was checked and achieved again (KMO value equal to 0.670; approximate chi-square value equal to 1242.767, with *p*-value < 0.001). The highest positive correlations were observed between Ba and Mn (0.892), Fe and Ca (0.889), and Cu and Zn (0.859). 

Six principal components with eigenvalues exceeding one (6.386, 4.168, 3.277, 2.296, 1.170, and 1.059) were extracted according to the Kaiser criterion. The extracted components explained up to 87.411% of the total variance (30.409%, 19.847%, 15.606%, 10.935%, 5.573%, and 5.041%, respectively). Variables with low saturation were not identified. 

The first component has the highest positive correlations with Zn (0.883) and Ni, and the highest negative correlation with Pb (−0.366). The dominant variables in the second component are B (−0.771), Fe (0.774), and Mo (0.719), while in the third component, they are Li (0.860) and Al (0.843); in the fourth they are Na (0.827) and As (0.786); in the fifth they are Cr (0.560) and Pb (0.550); and in the sixth component, the dominant variable is Se (0.583). 

**Figure 2:**
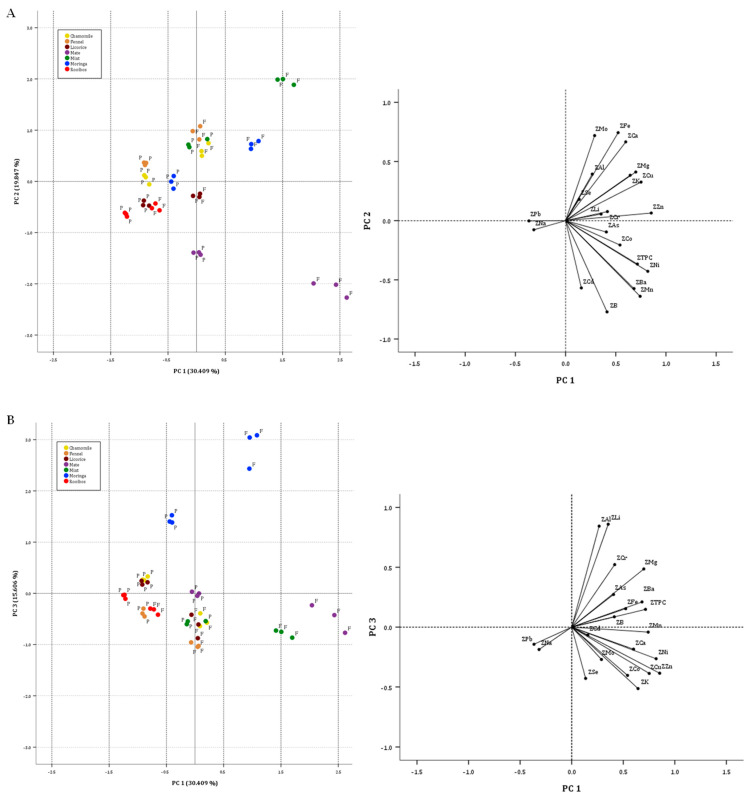
Two-dimensional score plots for the 42 herbal infusion samples categorized by botanical specie. ((**A**), PC2 against PC1 plot; (**B**), PC3 against PC1 plot; F, infusion obtained by filters; P, infusion obtained by pods). Insert: loading plot of minerals in the spaces defined by PCs.

As can be seen in Figure 2, the difference produced by the method of preparation of the infusion is highlighted for each of the plant materials used. When compared to Figure 1, the separation by plant material is still evident, but less pronounced. As can be seen, the samples obtained with the traditional preparation method have more positive PC1 values than those obtained with the coffee machine, and they are characterized by higher values of almost all the variables analyzed. In this way, the effects of the two types of infusion and their influences on the extraction of the minerals can be clearly seen.

### 3.6. Elemental Transfer Rates

The transfer rate of elements from herbal materials to herbal infusions is reported in Table 4. Overall, the results showed that the average transfer values for herbal infusions obtained by classic filter infusion were significantly higher than those obtained from pods using the coffee machine for almost all the elements studied. In the case of Pb and Cd, the result was the opposite, which is probably related to the different methods of preparation in terms of temperature, pressure, and time. There were no significant differences (*p* > 0.05) for Cr, Al, Ba, and Hg. 

Certainly, there are some exceptions, primarily in the case of moringa leaves, which showed higher values for almost all the percentages of transfer rate in the case of pods; other exceptions were observed for chamomile flowers, limited to Mn, Cr, Al, B, and Ba. Aluminum was also an exception for fennel seeds and licorice roots, and Cr was an exception for mate leaves. Other exceptions are rooibos leaves for Pb and Cd, and fennel seeds and licorice roots for Pb only.

The elements released in the infusions can be classified into three groups [25,60,61]. From the results obtained, in the case of filters, K, Mg, Ca, Na, Fe, Mn, Zn, Cu, Mo, Co, and Se can be classified as highly extractable elements (>55%), Cr, Al, B, Ba, Ni, Li, and As as moderately extractable elements (22–55%), and Pb, Cd, and Hg as poorly extractable elements (<20%), while, in the case of pods, all the elements can be classified as moderately extractable, except Li and As, which can be classified as poorly extractable elements. The highest release percentages of K, Mg, Ca, Na, Fe, Mn, Zn, Mo, Co, and Se were found in the mint leaf infusions obtained by filters. The highest release percentages of Cr, Al, B, Ba, and Pb were found in the infusions obtained using pods from chamomile flowers and moringa leaves. 

The highest transfer rate values were 95.8% for K, 93.5% for Mo, and 90.6% for Fe. These values were obtained for infusions of mint using the filter technique. Regarding herbal infusions obtained from pods, the highest transfer rates (with a maximum value of 79% for Mo and Co) for all elements were found in moringa and chamomile samples. 

From the results obtained, it can be confirmed that the transfer rate of elements depends on many factors, including the herbal material, the part of the plant used for infusion, and the preparation of the infusion method. Elements can form complexes with the organic compounds and be strongly bound to the matrix. This, combined with the different temperatures, times, and pressures used during the infusion preparation, can affect the solubility of elements and consequently result in highly variable transfer rates among different herbal infusions and between the two methods of preparations (traditional infusion and brewing using the coffee machine).

### 3.7. Polyphenols and Mineral Elemental Uptake by Herbal Infusions

The TPCs and the percentages of the reference values of mineral elements from the consumption of one cup (0.25 L) of herbal infusion analyzed in this study are reported in Table 5. The amounts of polyphenols were between 41.5 and 429 mg. Chamomile and fennel infusions from filters and pods were comparable, whereas mate, mint, moringa, and red rooibos infusions from filters had higher TPCs than those from pods. On the contrary, licorice infusions obtained from pods had higher TPC values than those obtained from filters. There are currently no EFSA-approved claims specifically for polyphenol products as substances that can protect blood lipids from the harmful effects of oxidative stress, with the exception of hydroxytyrosol and its derivatives in olive oil [62]. Given that the average total polyphenol content of herbal extract supplements is between 62 and 250 mg per capsule, consuming one cup (0.25 L) of each infusion analyzed provides amounts in this range, except for chamomile and fennel. In the case of licorice, only infusions made from the pods provide these amounts. In the case of mate and moringa, the values are even higher (429 and 299 mg, respectively).

Overall, the coverage of reference values was very low. The results for manganese are interesting: mate infusions by pods and filters cover 26.28% and 69.27% of the RDA, respectively, followed by licorice (10.08–18.27%), moringa (7.02–14.66%), mint (4.05–9.73%), and chamomile (3.81–6.10%) infusions.

On the other hand, moringa infusions were characterized by, in addition to their Mn content, the provision of more than 2% of Cr, Mg, Fe, and Al by infusion from filters, and of Mg and Al by infusion from filters. Meanwhile, mint infusions were characterized by, in addition to their Mn content, providing more than 2% of Ca, Mg, Mo, and Fe by infusion from filters.

The highest uptake of As, Pb, or Cd with the analyzed herbal infusions was 0.75% (value obtained in red rooibos infusions from filters).

## 4. Conclusions

Considering the growing consumption of herbal infusions among the population, the mineral profiles and the total polyphenol contents have been evaluated in various herbs and their respective infusions, obtained with two different methods of preparation. The data showed that the filtration method generally resulted in infusions with higher levels of polyphenols and mineral elements, except for cadmium and lead. In particular, mate tea prepared by the filtration method was found to have higher levels of manganese and polyphenols, providing consumers with a safe and beneficial health beverage option. Moreover, all infusions can be safely consumed with respect to As, Cd, Pb, and Hg. The PCA statistical model allowed us to discriminate the different types of herbal materials and highlighted the influences of the two types of infusion on the extraction of the minerals. However, to best characterize these products, the authors will focus their future attention on the qualitative and quantitative analysis of individual polyphenolic compounds in both the herbal materials and their infusions. 

## Figures and Tables

**Table 1:** Total polyphenol contents (TCPs, results expressed as gallic acid equivalents (GAEs)) in herbal materials of seven different plants, in their respective infusions and with their transfer rates % ((mg in herbal infusion/mg in herbal material) × 100).

Herbal Material	TPC in Herbal Material (mg GAE/g)	TPC in Herbal Infusion (mg GAE/g)	Transfer Rate (%)
			**Value based on herbal material**
*Flowers of* *Chamomile*			
In Pods	59.26 ± 3.34	42.09 ± 4.21	70.99 ± 5.27
In Filters	46.02 ± 3.87	41.44 ± 3.13	90.59 ± 11.46
***Average Value***	52.64 ± 7.94 (b) *	**41.76 ± 3.34 (b)**	
*Seeds of Fennel*			
In Pods	28.70 ± 2.45	15.37 ± 1.63	54.15 ± 10.45
In Filters	15.74 ± 1.85	13.45 ± 1.01	85.74 ± 4.16
***Average Value***	**22.22 ± 7.36 (a)**	**14.41 ± 1.61 (a)**	
*Roots of Licorice*			
In Pods	105.86 ± 8.60	60.26 ± 4.63	57.13 ± 5.73
In Filters	42.90 ± 3.25	18.64 ± 0.93	43.73 ± 5.46
***Average Value***	**74.38 ± 34.97 (bc)**	**39.45 ± 22.99 (b)**	
*Leaves of Mate*			
In Pods	141.67 ± 10.92	69.03 ± 5.44	48.73 ± 1.31
In Filters	176.54 ± 12.50	171.60 ± 14.97	97.13 ± 1.93
***Average Value***	**159.11 ± 21.80 (d)**	**120.32 ± 57.08 (d)**	
*Leaves of Mint*			
In Pods	96.39 ± 6.48	63.49 ± 5.96	65.96 ± 6.04
In Filters	109.88 ± 10.24	101.85 ± 9.26	93.44 ± 13.86
***Average Value***	**103.13 ± 10.65 (c)**	**82.67 ± 22.14 (c)**	
*Leaves of Moringa*			
In Pods	81.79 ± 7.43	40.43 ± 3.13	49.94 ± 8.33
In Filters	111.11 ± 11.23	99.79 ± 9.43	90.09 ± 8.17
***Average Value***	**96.45 ± 18.18 (c)**	**70.11 ± 33.11 (c)**	
*Leaves of Red Rooibos*			
In Pods	107.41 ± 9.76	61.83 ± 6.18	57.54 ± 0.85
In Filters	158.64 ± 10.65	86.42 ± 9.26	54.38 ± 2.20
***Average Value***	**133.02 ± 29.51 (d)**	**74.13 ± 15.19 (c)**	
Average Value for Pods			57.78 ± 9.36
Average Value for Filters			79.30 ± 21.17 *I* **

* Statistical results for different herbal materials obtained by Kruskal–Wallis test. Different letters in the same column represent statistically different results (*p* < 0.05). ** Statistical results for different bags obtained by Mann–Whitney U test. *I* indicates statistically higher results (*p* < 0.05).

**Table 2:** Elemental contents in herbal materials of seven different plants.

**Herbal Materials**	**K** **(g/kg)**	**Mg** **(g/kg)**	**Ca** **(g/kg)**	**Na** **(mg/kg)**	**Fe** **(mg/kg)**		**Mn** **(mg/kg)**		**Zn** **(mg/kg)**		**Cu** **(mg/kg)**	**Cr** **(mg/kg)**		**Mo** **(mg/kg)**		**Co** **(mg/kg)**		**Se** **(mg/kg)**
*Flowers of Chamomile*																		
In Pods	9.69 ± 2.36	3.63 ± 0.19	2.17 ± 0.18	144.59 ± 5.66	193.21 ± 3.77		81.39 ± 3.84		23.39 ± 0.36		6.78 ± 0.53	1.12 ± 0.46		0.78 ± 0.24		0.21 ± 0.04		0.15 ± 0.04
In Filters	10.34 ± 2.84	4.18 ± 0.03	2.27 ± 0.09	129.49 ± 2.31	159.42 ± 10.21		79.51 ± 7.22		19.78 ± 0.65		4.94 ± 0.16	1.00 ± 0.14		0.95 ± 0.34		0.22 ± 0.03		0.20 ± 0.06
***Average Value***	**10.02 ± 2.36** (b) *	**3.90 ± 0.33** **(b)**	**2.22 ± 0.14** **(a)**	**137.02 ± 9.13** **(a)**	**176.33 ± 19.75** **(c)**		**80.45 ± 5.27** **(b)**		**21.58 ± 2.03** **(c)**		**5.86 ± 1.07** **(b)**	**1.06 ± 0.31** **(d)**		**0.87 ± 0.28** **(c)**		**0.21 ± 0.03** **(ab)**		**0.18 ± 0.05** **(b)**
*Seeds of Fennel*																		
In Pods	17.00 ± 4.10	4.53 ± 0.20	5.13 ± 0.21	758.58 ± 6.97	169.31 ± 13.99		48.84 ± 6.96		23.30 ± 1.10		10.47 ± 0.27	0.34 ± 0.11		0.23 ± 0.18		0.10 ± 0.02		0.23 ± 0.07
In Filters	20.48 ± 7.12	5.98 ± 0.09	5.38 ± 0.20	862.11 ± 10.20	142.52 ± 9.45		62.19 ± 8.20		21.31 ± 0.69		7.59 ± 0.17	0.23 ± 0.08		0.30 ± 0.18		0.12 ± 0.03		0.17 ± 0.05
***Average Value***	**18.74 ± 5.53** **(c)**	**5.26 ± 0.81** **(c)**	**5.26 ± 0.22** **(c)**	**810.35 ± 57.23** **(d)**	**155.90 ± 18.15** **(bc)**		**55.52 ± 9.98** **(b)**		**22.58 ± 1.37** **(c)**		**9.03 ± 1.59** **(c)**	**0.28 ± 0.10** **(a)**		**0.27 ± 0.16** **(b)**		**0.11 ± 0.02** **(a)**		**0.20 ± 0.06** **(b)**
*Roots of Licorice*																		
In Pods	9.84 ± 3.00	3.53 ± 0.12	2.17 ± 0.19	355.43 ± 11.80	125.01 ± 7.46		236.67 ± 8.70		10.13 ± 0.49		2.50 ± 0.20	0.68 ± 0.10		0.20 ± 0.06		0.20 ± 0.01		0.10 ± 0.04
In Filters	10.94 ± 3.04	4.25 ± 0.04	2.22 ± 0.08	431.78 ± 3.77	94.30 ± 8.99		223.60 ± 26.94		14.57 ± 0.71		3.94 ± 0.14	0.61 ± 0.12		0.19 ± 0.09		0.39 ± 0.04		0.11 ± 0.03
***Average value***	**10.39 ± 2.77** **(b)**	**3.89 ± 0.40** **(b)**	**2.19 ± 0.13** **(a)**	**393.60 ± 57.23** **(c)**	**109.65 ± 18.36** **(b)**		**230.13 ± 19.29** **(d)**		**12.35 ± 2.49** **(b)**		**3.22 ± 0.81** **(ab)**	**0.65 ± 0.11** **(c)**		**0.19 ± 0.07** **(b)**		**0.29 ± 0.10** **(b)**		**0.10 ± 0.03** **(a)**
*Leaves of Mate*																		
In Pods	12.17 ± 5.46	4.22 ± 0.37	3.36 ± 0.17	95.12 ± 6.31	7.51 ± 1.20		623.54 ± 4.06		35.12 ± 0.69		8.18 ± 0.28	0.85 ± 0.11		0.04 ± 0.02		0.38 ± 0.05		0.04 ± 0.02
In Filters	14.44 ± 5.10	5.63 ± 0.07	3.22 ± 0.10	105.92 ± 2.04	12.15 ± 0.80		889.60 ± 25.18		35.69 ± 1.19		9.00 ± 0.14	0.97 ± 0.13		0.02 ± 0.01		0.32 ± 0.04		0.12 ± 0.04
***Average Value***	**13.30 ± 4.89** **(b)**	**4.92 ± 0.81** **(bc)**	**3.29 ± 0.15** **(b)**	**100.52 ± 7.23** **(a)**	**9.85 ± 2.70** **(a)**		**756.57 ± 146.62** **(e)**		**35.41 ± 0.92** **(d)**		**8.59 ± 0.49** **(c)**	**0.91 ± 0.13** **(cd)**		**0.03 ± 0.02** **(a)**		**0.35 ± 0.05** **(c)**		**0.08 ± 0.05** **(a)**
*Leaves of Mint*																		
In Pods	19.27 ± 6.80	7.00 ± 0.02	12.19 ± 0.49	278.66 ± 2.44	890.72 ± 22.44		100.52 ± 10.09		42.00 ± 0.98		17.70 ± 0.16	1.12 ± 0.15		0.95 ± 0.34		0.31 ± 0.04		0.07 ± 0.03
In Filters	17.94 ± 5.98	7.50 ± 0.02	13.14 ± 0.43	303.99 ± 2.92	815.63 ± 27.44		121.44 ± 15.23		35.44 ± 1.02		12.66 ± 0.18	1.22 ± 0.16		1.01 ± 0.38		0.25 ± 0.02		0.10 ± 0.04
***Average Value***	**18.61 ± 5.77** **(c)**	**7.25 ± 0.27** **(d)**	**12.67 ± 0.67** **(d)**	**291.32 ± 14.07** **(b)**	**853.17 ± 46.83** **(e)**		**110.98 ± 16.28** **(c)**		**38.72 ± 3.70** **(d)**		**15.18 ± 2.77** **(d)**	**1.17 ± 0.15** **(d)**		**0.98 ± 0.32** **(c)**		**0.28 ± 0.04** **(b)**		**0.08 ± 0.04** **(a)**
*Leaves of Moringa*																		
In Pods	5.61 ± 1.86	10.89 ± 0.18	4.35 ± 0.24	270.33 ± 6.50	490.68 ± 8.13		157.60 ± 8.26		12.48 ± 0.43		5.81 ± 0.29	0.80 ± 0.13		0.31 ± 0.17		0.17 ± 0.03		0.09 ± 0.03
In Filters	6.00 ± 2.11	10.72 ± 0.50	4.25 ± 0.14	241.52 ± 2.21	508.73 ± 18.29		179.69 ± 20.11		12.00 ± 0.41		3.75 ± 0.13	0.95 ± 0.12		0.21 ± 0.11		0.09 ± 0.01		0.06 ± 0.02
***Average Value***	**5.81 ± 1.79** **(a)**	**10.81 ± 0.35** **(e)**	**4.30 ± 0.18** **(c)**	**255.93 ± 16.37** **(b)**	**499.70 ± 16.07** **(d)**		**168.65 ± 18.31** **(c)**		**12.24 ± 0.46** **(b)**		**4.78 ± 1.15** **(b)**	**0.88 ± 0.14** **(cd)**		**0.26 ± 0.14** **(b)**		**0.13 ± 0.05** **(a)**		**0.08 ± 0.03** **(a)**
*Leaves of Red Rooibos*																		
In Pods	3.06 ± 1.33	1.96 ± 0.04	1.38 ± 0.16	2965.45 ± 33.57	7.77 ± 0.63		20.58 ± 4.23		6.93 ± 0.18		1.54 ± 0.37	0.44 ± 0.06		0.16 ± 0.04		0.20 ± 0.03		0.17 ± 0.04
In Filters	3.55 ± 0.94	2.41 ± 0.02	1.86 ± 0.06	2514.33 ± 6.23	10.33 ± 0.68		29.04 ± 5.30		8.57 ± 0.38		2.20 ± 0.12	0.55 ± 0.10		0.22 ± 0.12		0.20 ± 0.02		0.14 ± 0.05
***Average Value***	**3.30 ± 1.07** **(a)**	**2.19 ± 0.25** **(a)**	**1.62 ± 0.28** **(a)**	**2739.90 ± 248.04** **(e)**	**9.05 ± 1.52** **(a)**		**24.81 ± 6.31** **(a)**		**7.75 ± 0.84** **(a)**		**1.87 ± 0.44** **(a)**	**0.49 ± 0.09** **(bc)**		**0.19 ± 0.09** **(b)**		**0.20 ± 0.03** **(ab)**		**0.16 ± 0.04** **(b)**
**Herbal Materials**	**Al** **(mg/kg)**	**B** **(mg/kg)**		**Ba** **(mg/kg)**		**Ni** **(mg/kg)**		**Li** **(mg/kg)**		**As** **(mg/kg)**			**Pb** **(mg/kg)**		**Cd** **(mg/kg)**		**Hg** **(mg/kg)**	
*Flowers of Chamomile*																		
In Pods	202.78 ± 6.95	28.33 ± 0.87		1.02 ± 0.26		0.82 ± 0.42		0.044 ± 0.014		0.089 ± 0.024			0.097 ± 0.023		0.057 ± 0.013		<LOQ	
In Filters	178.37 ± 9.13	23.22 ± 0.77		2.94 ± 0.81		1.02 ± 0.58		0.032 ± 0.005		0.066 ± 0.045			0.042 ± 0.022		0.135 ± 0.031		<LOQ	
***Average Value***	**190.58 ± 15.24** **(cd)**	**25.78 ± 2.90** **(bc)**		**1.98 ± 1.18** **(a)**		**0.92 ± 0.47** **(a)**		**0.038 ± 0.011** **(b)**		**0.077 ± 0.035** **(ab)**			**0.070 ± 0.037**		**0.096 ± 0.048** **(b)**		**<LOQ** **(a)**	
*Seeds of Fennel*																		
In Pods	167.94 ± 10.91	17.71 ± 0.87		1.09 ± 0.44		1.69 ± 0.95		0.061 ± 0.014		0.059 ± 0.029			0.045 ± 0.018		0.005 ± 0.001		<LOQ	
In Filters	155.33 ± 8.20	15.36 ± 0.66		1.89 ± 0.71		1.03 ± 0.60		0.046 ± 0.005		0.033 ± 0.012			0.066 ± 0.024		0.006 ± 0.001		<LOQ	
***Average Value***	**161.63 ± 11.08** **(c)**	**16.54 ± 1.46** **(b)**		**1.49 ± 0.69** **(a)**		**1.36 ± 0.80** **(a)**		**0.054 ± 0.013** **(b)**		**0.046 ± 0.025** **(a)**			**0.056 ± 0.022**		**0.006 ± 0.001** **(a)**		**<LOQ** **(a)**	
*Roots of Licorice*																		
In Pods	67.56 ± 11.55	17.54 ± 0.68		23.61 ± 0.18		0.80 ± 0.36		<LOQ		0.077 ± 0.029			0.018 ± 0.006		0.041 ± 0.012		<LOQ	
In Filters	59.54 ± 4.99	21.31 ± 0.80		19.39 ± 0.90		0.92 ± 0.50		<LOQ		0.052 ± 0.039			0.046 ± 0.030		0.008 ± 0.001		<LOQ	
***Average Value***	**63.55 ± 9.11** **(b)**	**19.43 ± 2.17** **(b)**		**21.50 ± 2.39** **(d)**		**0.86 ± 0.40** **(a)**		**<LOQ** **(a)**		**0.064 ± 0.034** **(ab)**			**0.032 ± 0.025**		**0.024 ± 0.020** **(a)**		**<LOQ** **(a)**	
*Leaves of Mate*																		
In Pods	52.05 ± 6.41	50.30 ± 0.78		20.86 ± 0.79		3.45 ± 0.68		0.062 ± 0.016		0.071 ± 0.037			0.013 ± 0.008		0.293 ± 0.017		0.006 ± 0.002	
In Filters	64.33 ± 5.22	56.99 ± 2.03		34.33 ± 1.33		2.99 ± 0.60		0.078 ± 0.009		0.102 ± 0.043			0.040 ± 0.019		0.153 ± 0.022		0.005 ± 0.002	
***Average Value***	**58.20 ± 8.52** **(b)**	**53.65 ± 3.91** **(e)**		**27.60 ± 7.44** **(e)**		**3.22 ± 0.63** **(b)**		**0.070 ± 0.014** **(b)**		**0.087 ± 0.040** **(ab)**			**0.026 ± 0.019**		**0.223 ± 0.078** **(c)**		**0.006 ± 0.001** **(b)**	
*Leaves of Mint*																		
In Pods	215.22 ± 5.12	3.45 ± 0.50		12.31 ± 0.37		1.23 ± 0.37		<LOQ		0.105 ± 0.039			0.098 ± 0.038		0.049 ± 0.012		0.010 ± 0.002	
In Filters	201.01 ± 4.88	4.11 ± 0.71		10.98 ± 0.45		1.43 ± 0.45		<LOQ		0.087 ± 0.027			0.016 ± 0.002		0.004 ± 0.001		0.006 ± 0.001	
***Average Value***	**208.13 ± 8.97** **(d)**	**3.78 ± 0.66** **(a)**		**11.65 ± 0.81** **(c)**		**1.33 ± 0.385** **(a)**		**<LOQ** **(a)**		**0.096 ± 0.031** **(b)**			**0.057 ± 0.051**		**0.026 ± 0.026** **(a)**		**0.008 ± 0.002** **(b)**	
*Leaves of Moringa*																		
In Pods	593.59 ± 10.32	35.01 ± 0.70		19.68 ± 0.88		0.75 ± 0.41		0.521 ± 0.067		0.101 ± 0.039			0.049 ± 0.014		0.021 ± 0.007		0.007 ± 0.002	
In Filters	601.23 ± 10.45	29.89 ± 0.92		16.29 ± 0.75		0.87 ± 0.49		0.331 ± 0.066		0.112 ± 0.055			0.018 ± 0.003		0.003 ± 0.001		0.004 ± 0.001	
***Average Value***	**597.40 ± 10.19** **(e)**	**32.45 ± 2.90** **(c)**		**17.99 ± 1.99** **(d)**		**0.81 ± 0.41** **(a)**		**0.426 ± 0.120** **(c)**		**0.106 ± 0.043** **(b)**			**0.034 ± 0.019**		**0.012 ± 0.010** **(a)**		**0.006 ± 0.002** **(b)**	
*Leaves of Red Rooibos*																		
In Pods	5.11 ± 0.51	42.33 ± 0.64		7.05 ± 0.39		1.00 ± 0.27		0.031 ± 0.023		0.113 ± 0.041			0.045 ± 0.015		0.048 ± 0.011		<LOQ	
In Filters	7.11 ± 1.21	46.78 ± 1.78		5.15 ± 1.99		0.89 ± 0.43		0.017 ± 0.004		0.151 ± 0.077			0.097 ± 0.049		0.058 ± 0.011		<LOQ	
***Average Value***	**6.10 ± 1.38** **(a)**	**44.56 ± 2.71** **(d)**		**6.10 ± 1.66** **(b)**		**0.95 ± 0.33** **(a)**		**0.024 ± 0.016** **(b)**		**0.132 ± 0.059** **(b)**			**0.071 ± 0.043**		**0.053 ± 0.011** **(ab)**		**<LOQ** **(a)**	

* Statistical results for different plant materials obtained by Kruskal–Wallis test. Different letters in the same column represent statistically different results (*p* < 0.05).

**Table 3:** Elemental contents in herbal infusions of seven different herbal materials.

**Herbal Materials**	**K** **(mg/L)**		**Mg** **(mg/L)**		**Ca** **(mg/L)**		**Na** **(mg/L)**	**Fe** **(mg/L)**		**Mn** **(mg/L)**		**Zn** **(µg/L)**		**Cu** **(µg/L)**	**Cr** **(µg/L)**		**Mo** **(µg/L)**		**Co** **(µg/L)**		**Se** **(µg/L)**
*Flowers of Chamomile*																					
In Pods	25.92 ± 0.89		10.45 ± 0.42		4.76 ± 0.54		0.39 ± 0.06	0.16 ± 0.02		0.31 ± 0.04		52.20 ± 3.65		19.51 ± 2.13	3.60 ± 0.44		2.22 ± 0.09		0.22 ± 0.07		0.38 ± 0.12
In Filters	67.85 ± 1.12		25.99 ± 1.11		12.06 ± 1.34		0.83 ± 0.10	0.97 ± 0.15		0.49 ± 0.07		107.42 ± 14.06		31.94 ± 6.50	2.94 ± 0.33		5.97 ± 0.76		1.20 ± 0.22		1.23 ± 0.18
***Average Value***	**46.89 ± 22.98** (ab) *		**18.22 ± 8.55** **(b)**		**8.41 ± 4.10** **(a)**		**0.61 ± 0.25** **(a)**	**0.57 ± 0.46** **(b)**		**0.40 ± 0.11** **(a)**		**79.67 ± 31.63** **(b)**		**25.72 ± 8.07** **(c)**	**3.27 ± 0.50** **(d)**		**4.08 ± 2.12** **(c)**		**0.70 ± 0.53** **(a)**		**0.80 ± 4.87** **(b)**
*Seeds of Fennel*																					
In Pods	44.16 ± 1.20		14.20 ± 0.58		16.31 ± 0.94		2.18 ± 0.13	0.35 ± 0.06		0.17 ± 0.03		57.90 ± 5.53		26.59 ± 2.23	0.50 ± 0.09		0.77 ± 0.14		0.35 ± 0.09		0.63 ± 0.13
In Filters	134.81 ± 3.00		37.41 ± 1.34		32.37 ± 2.00		5.55 ± 0.14	0.92 ± 0.27		0.38 ± 0.09		117.06 ± 20.72		46.82 ± 16.37	0.59 ± 0.14		1.70 ± 0.44		0.65 ± 0.20		1.04 ± 0.16
***Average Value***	**89.49 ± 49.69** **(b)**		**25.81 ± 12.74** **(bc)**		**24.34 ± 8.90** **(b)**		**3.86 ± 1.85** **(c)**	**0.64 ± 0.36** **(b)**		**0.28 ± 0.13** **(a)**		**87.17 ± 35.06** **(b)**		**36.68 ± 15.23** **(c)**	**0.53 ± 0.15** **(a)**		**1.23 ± 0.59** **(b)**		**0.52 ± 0.21** **(a)**		**0.83 ± 0.26** **(b)**
*Roots of Licorice*																					
In Pods	22.53 ± 0.80		10.75 ± 0.44		7.66 ± 0.80		1.13 ± 0.12	0.20 ± 0.04		0.81 ± 0.11		22.50 ± 2.00		7.81 ± 0.90	1.16 ± 0.23		0.36 ± 0.07		0.61 ± 0.16		0.13 ± 0.06
In Filters	70.35 ± 1.34		26.54 ± 1.00		13.36 ± 1.01		2.75 ± 0.17	0.61 ± 0.22		1.46 ± 0.09		88.15 ± 13.65		22.88 ± 6.07	1.55 ± 0.51		1.13 ± 0.30		2.45 ± 0.53		0.59 ± 0.12
***Average Value***	**46.44 ± 26.21** **(ab)**		**18.65 ± 8.68** **(b)**		**10.51 ± 3.23** **(a)**		**1.94 ± 0.90** **(b)**	**0.40 ± 0.26** **(b)**		**1.13 ± 0.37** **(b)**		**55.17 ± 37.03** **(ab)**		**15.35 ± 9.11** **(b)**	**1.35 ± 0.40** **(b)**		**0.72 ± 0.46** **(ab)**		**1.55 ± 1.06** **(b)**		**0.36 ± 0.26** **(a)**
*Leaves of Mate*																					
In Pods	33.65 ± 1.24		12.46 ± 0.55		11.31 ± 0.98		0.31 ± 0.05	0.02 ± 0.00		2.10 ± 0.14		79.22 ± 6.00		14.34 ± 1.20	2.46 ± 0.35		0.09 ± 0.03		1.26 ± 0.33		0.07 ± 0.03
In Filters	93.18 ± 2.60		36.08 ± 1.41		19.85 ± 1.50		0.68 ± 0.07	0.07 ± 0.02		5.54 ± 0.30		220.65 ± 28.61		58.55 ± 19.23	2.28 ± 0.37		0.14 ± 0.03		1.78 ± 0.65		0.69 ± 0.14
***Average Value***	**63.42 ± 32.66** **(b)**		**24.27 ± 12.97** **(bc)**		**15.58 ± 4.81** **(ab)**		**0.49 ± 0.21** **(a)**	**0.05 ± 0.03** **(a)**		**3.82 ± 1.89** **(c)**		**149.83 ± 79.72** **(c)**		**36.43 ± 27.10** **(c)**	**2.37 ± 0.34** **(c)**		**0.13 ± 0.05** **(a)**		**1.53 ± 0.55** **(b)**		**0.38 ± 0.35** **(a)**
*Leaves of Mint*																					
In Pods	61.37 ± 1.43		22.56 ± 0.81		35.21 ± 1.77		0.62 ± 0.10	1.90 ± 0.23		0.32 ± 0.06		151.92 ± 12.91		45.59 ± 3.52	1.31 ± 0.12		2.76 ± 0.44		0.89 ± 0.31		0.23 ± 0.07
In Filters	115.35 ± 3.23		45.46 ± 1.40		71.96 ± 3.21		1.99 ± 0.08	5.37 ± 0.44		0.78 ± 0.11		165.14 ± 28.55		62.97 ± 13.36	2.51 ± 0.52		6.37 ± 0.70		1.58 ± 0.52		0.46 ± 0.13
***Average Value***	**88.36 ± 29.66** **(b)**		**34.01 ± 12.58** **(c)**		**53.59 ± 20.26** **(c)**		**1.30 ± 0.75** **(b)**	**3.64 ± 1.92** **(d)**		**0.55 ± 0.26** **(a)**		**158.50 ± 20.81** **(c)**		**54.27 ± 12.91** **(d)**	**1.92 ± 0.75** **(b)**		**4.55 ± 2.06** **(c)**		**1.22 ± 0.53** **(b)**		**0.34 ± 0.16** **(a)**
*Leaves of Moringa*																					
In Pods	15.12 ± 0.43		36.72 ± 2.02		12.56 ± 0.83		0.66 ± 0.07	0.58 ± 0.07		0.56 ± 0.08		26.37 ± 1.12		15.76 ± 0.98	2.35 ± 0.23		0.94 ± 0.22		0.62 ± 0.21		0.14 ± 0.04
In Filters	39.27 ± 0.67		70.66 ± 1.63		27.40 ± 2.04		1.60 ± 0.08	3.35 ± 0.55		1.17 ± 0.13		76.92 ± 16.06		23.36 ± 7.28	3.63 ± 0.63		1.29 ± 0.40		0.52 ± 0.14		0.37 ± 0.12
***Average Value***	**27.19 ± 13.24** **(a)**		**53.69 ± 18.67** **(d)**		**19.98 ± 8.25** **(ab)**		**1.13 ± 0.52** **(b)**	**1.97 ± 1.56** **(c)**		**0.87 ± 0.35** **(ab)**		**51.83 ± 29.40** **(ab)**		**19.57 ± 6.23** **(b)**	**3.02 ± 0.83** **(d)**		**1.10 ± 0.35** **(bc)**		**0.57 ± 0.16** **(a)**		**0.26 ± 0.15** **(a)**
*Leaves of Red Rooibos*																					
In Pods	6.69 ± 0.31		5.13 ± 0.35		3.71 ± 0.41		7.94 ± 0.19	0.02 ± 0.01		0.06 ± 0.01		10.14 ± 1.74		2.54 ± 0.27	0.72 ± 0.12		0.18 ± 0.03		0.50 ± 0.13		0.41 ± 0.14
In Filters	23.34 ± 0.87		14.22 ± 1.13		11.41 ± 1.26		16.6 ± 0.09	0.06 ± 0.02		0.17 ± 0.03		43.46 ± 7.42		13.55 ± 2.30	1.06 ± 0.15		1.24 ± 0.27		1.00 ± 0.23		0.88 ± 0.20
***Average Value***	**15.02 ± 9.14** **(a)**		**9.68 ± 5.04** **(a)**		**7.56 ± 4.30** **(a)**		**12.26 ± 4.74** **(d)**	**0.04 ± 0.02** **(a)**		**0.11 ± 0.06** **(a)**		**26.67 ± 18.94** **(a)**		**8.03 ± 6.20** **(a)**	**0.88 ± 0.22** **(a)**		**0.73 ± 0.61** **(b)**		**0.77 ± 0.35** **(a)**		**0.64 ± 0.30** **(b)**
**Herbal Materials**		**Al** **(mg/L)**		**B** **(µg/L)**		**Ba** **(µg/L)**			**Ni** **(µg/L)**		**Li** **(µg/L)**		**As** **(µg/L)**			**Pb** **(µg/L)**		**Cd** **(µg/L)**		**Hg** **(µg/L)**	
*Flowers of Chamomile*																					
In Pods		0.45 ± 0.12		92.03 ± 36.96		3.73 ± 0.29			1.66 ± 0.28		0.019 ± 0.002		0.073 ± 0.028			0.232 ± 0.064		0.07 2 ± 0.030		<LOQ	
In Filters		0.52 ± 0.13		100.06 ± 3.55		10.59 ± 3.24			4.44 ± 0.15		0.076 ± 0.014		0.211 ± 0.073			0.080 ± 0.029		0.156 ± 0.045		<LOQ	
***Average Value***		**0.48 ± 0.12** **(b)**		**96.00 ± 24.07** **(c)**		**7.15 ± 4.27** **(a)**			**3.05 ± 1.52** **(a)**		**0.048 ± 0.032** **(a)**		**0.143 ± 0.091**			**0.155 ± 0.058** **(b)**		**0.115 ± 0.058** **(b)**		**<LOQ**	
*Seeds of Fennel*																					
In Pods		0.46 ± 0.07		39.73 ± 7.35		3.23 ± 0.22			2.40 ± 0.27		<LOQ		0.023 ± 0.006			0.047 ± 0.008		<LOQ		<LOQ	
In Filters		0.36 ± 0.07		70.22 ± 1.60		7.49 ± 2.81			3.33 ± 0.52		0.138 ± 0.047		0.096 ± 0.015			0.084 ± 0.014		<LOQ		<LOQ	
***Average Value***		**0.41 ± 0.08** **(b)**		**54.83 ± 17.24** **(b)**		**5.35 ± 2.92** **(a)**			**2.85 ± 0.62** **(a)**		**0.070 ± 0.081** **(ab)**		**0.060 ± 0.041**			**0.064 ± 0.023** **(a)**		**<LOQ** **(a)**		**<LOQ**	
*Roots of Licorice*																					
In Pods		0.18 ± 0.02		47.74 ± 12.45		48.78 ± 7.50			1.21 ± 0.25		<LOQ		0.073 ± 0.029			0.027 ± 0.002		0.131 ± 0.045		<LOQ	
In Filters		0.07 ± 0.01		99.21 ± 1.96		80.07 ± 6.32			3.34 ± 0.32		<LOQ		0.144 ± 0.037			0.076 ± 0.027		<LOQ		<LOQ	
***Average value***		**0.13 ± 0.06** **(a)**		**73.33 ± 29.16** **(bc)**		**64.42 ± 18.22** **(d)**			**2.25 ± 1.22** **(a)**		**<LOQ** **(a)**		**0.110 ± 0.050**			**0.052 ± 0.032** **(a)**		**0.066 ± 0.076** **(a)**		**<LOQ**	
*Leaves of Mate*																					
In Pods		0.16 ± 0.05		124.89 ± 21.15		62.63 ± 7.09			7.55 ± 0.42		0.051 ± 0.012		0.065 ± 0.026			0.021 ± 0.002		0.741 ± 0.070		<LOQ	
In Filters		0.10 ± 0.02		308.91 ± 2.01		162.57 ± 9.23			15.40 ± 0.44		0.300 ± 0.127		0.387 ± 0.072			0.062 ± 0.010		0.273 ± 0.088		<LOQ	
***Average Value***		**0.13 ± 0.04** **(a)**		**217.00 ± 0.10** **(e)**		**112.60 ± 55.23** **(e)**			**11.48 ± 4.31** **(c)**		**0.173 ± 0.161** **(b)**		**0.227 ± 0.185**			**0.040 ± 0.023** **(a)**		**0.507 ± 0.266** **(c)**		**<LOQ**	
*Leaves of Mint*																					
In Pods		0.49 ± 0.11		6.66 ± 1.28		40.93 ± 4.48			2.46 ± 0.35		<LOQ		0.097 ± 0.032			0.267 ± 0.079		0.141 ± 0.040		<LOQ	
In Filters		0.60 ± 0.12		11.83 ± 0.82		33.35 ± 4.85			7.66 ± 1.34		<LOQ		0.238 ± 0.060			<LOQ		<LOQ		<LOQ	
***Average Value***		**0.55 ± 0.12** **(b)**		**9.17 ± 2.86** **(a)**		**37.13 ± 5.91** **(c)**			**5.05 ± 2.99** **(b)**		**<LOQ** **(a)**		**0.167 ± 0.088**			**0.134 ± 0.155** **(b)**		**0.071 ± 0.080** **(a)**		**<LOQ**	
*Leaves of Moringa*																					
In Pods		1.15 ± 0.17		81.99 ± 24.91		71.86 ± 11.77			1.15 ± 0.12		0.591 ± 0.100		0.128 ± 0.041			0.100 ± 0.028		0.073 ± 0.018		<LOQ	
In Filters		2.87 ± 0.17		123.52 ± 2.21		72.67 ± 3.16			3.18 ± 0.70		1.044 ± 0.299		0.513 ± 0.101			0.031 ± 0.004		<LOQ		<LOQ	
***Average Value***		**2.01 ± 0.96** **(c)**		**102.27 ± 27.52** **(c)**		**72.25 ± 7.72** **(d)**			**2.17 ± 1.19** **(a)**		**0.818 ± 0.316** **(c)**		**0.320 ± 0.223**			**0.063 ± 0.041** **(a)**		**0.037 ± 0.041** **(a)**		**<LOQ**	
*Leaves of Red Rooibos*																					
In Pods		0.01 ± 0.00		141.31 ± 17.18		17.33 ± 2.65			2.89 ± 0.32		<LOQ		0.116 ± 0.032			0.115 ± 0.032		0.019 ± 0.002		<LOQ	
In Filters		0.01 ± 0.00		146.12 ± 1.49		20.52 ± 5.87			2.64 ± 0.20		0.031 ± 0.004		0.633 ± 0.133			0.231 ± 0.067		0.044 ± 0.004		<LOQ	
***Average Value***		**0.01 ± 0.00** **(a)**		**144.00 ± 11.19** **(d)**		**18.93 ± 4.43** **(b)**			**2.77 ± 2.88** **(a)**		**0.016 ± 0.016** **(a)**		**0.373 ± 0.298**			**0.172 ± 0.081** **(b)**		**0.033 ± 0.015** **(a)**		**<LOQ**	

* Statistical results for different herbal infusions obtained by Kruskal–Wallis test. Different letters in the same column represent statistically different results (*p* < 0.05).

**Table 4:** Transfer rates (%) of elements from herbal materials to herbal infusions.

**Herbal Materials**	**K**	**Mg**		**Ca**	**Na**	**Fe**		**Mn**		**Zn**		**Cu**		**Cr**	**Mo**		**Co**		**Se**
*Flowers of Chamomile*																			
In Pods	55.1 ± 17.6	56.4 ± 5.1		42.8 ± 3.0	53.2 ± 10.5	16.1 ± 2.3		73.3 ± 11.3		43.6 ± 2.9		56.3 ± 6.2		71.9 ± 36.6	59.1 ± 20.3		21.7 ± 10.4		53.0 ± 27.0
In Filters	67.4 ± 19.5	60.6 ± 2.9		51.8 ± 5.2	62.7 ± 8.1	60.1 ± 12.9		59.7 ± 5.0		52.8 ± 5.4		62.8 ± 11.1		29.0 ± 6.3	67.4 ± 29.7		54.5 ± 11.5		61.3 ± 8.3
*Seeds of Fennel*																			
In Pods	24.1 ± 5.6	28.0 ± 1.5		28.4 ± 0.54	25.7 ± 1.5	18.8 ± 3.5		31.4 ± 4.3		22.2 ± 1.9		22.7 ± 1.8		13.7 ± 2.4	46.2 ± 34.7		31.6 ± 12.9		28.0 ± 15.7
In Filters	62.0 ± 21.4	54.2 ± 2.4		52.3 ± 5.2	55.8 ± 1.6	56.5 ± 18.7		54.5 ± 17.6		47.5 ± 6.8		53.6 ± 19.6		23.1 ± 5.6	56.2 ± 21.0		45.9 ± 4.2		58.2 ± 23.9
*Roots of Licorice*																			
In Pods	25.4 ± 8.7	31.5 ± 0.8		36.8 ± 5.9	32.8 ± 4.4	16.5 ± 2.5		35.2 ± 4.4		23.0 ± 2.4		32.7 ± 6.1		17.9 ± 3.9	19.9 ± 8.1		31.1 ± 6.9		16.5 ± 9.7
In Filters	69.2 ± 21.9	63.4 ± 2.0		61.1 ± 6.0	64.5 ± 3.6	64.3 ± 17.1		66.6 ± 4.7		61.4 ± 9.4		58.5 ± 13.4		25.8 ± 8.2	65.8 ± 14.3		65.5 ± 19.2		55.6 ± 12.9
*Leaves of Mate*																			
In Pods	26.2 ± 12.0	24.3 ± 1.5		27.7 ± 3.9	26.3 ± 4.4	22.5 ± 4.2		27.6 ± 1.6		18.5 ± 1.1		14.4 ± 1.7		23.8 ± 0.5	20.0 ± 11.4		27.5 ± 8.8		15.2 ± 3.0
In Filters	56.0 ± 20.8	50.9 ± 1.4		48.8 ± 2.2	51.2 ± 6.5	45.0 ± 11.1		49.4 ± 1.8		49.0 ± 4.8		51.8 ± 17.6		19.1 ± 4.5	46.3 ± 6.0		44.4 ± 16.4		47.8 ± 16.9
*Leaves of Mint*																			
In Pods	40.5 ± 14.9	37.4 ± 1.2		33.6 ± 1.2	25.8 ± 4.2	24.8 ± 2.3		37.3 ± 2.9		42.1 ± 4.5		29.9 ± 2.1		13.9 ± 2.7	35.3 ± 6.5		33.4 ± 7.4		42.2 ± 20.4
In Filters	95.8 ± 33.3	83.4 ± 2.7		75.3 ± 2.1	89.9 ± 3.2	90.6 ± 6.2		88.1 ± 2.9		64.0 ± 9.4		68.6 ± 15.3		28.2 ± 5.1	93.5 ± 27.6		88.0 ± 32.1		77.2 ± 39.1
*Leaves of Moringa*																			
In Pods	63.5 ± 22.4	73.3 ± 3.8		62.8 ± 1.6	53.3 ± 5.4	25.8 ± 3.2		77.9 ± 14.2		46.0 ± 3.5		58.9 ± 0.7		65.5 ± 17.9	79.4 ± 42.7		79.2 ± 12.3		41.5 ± 29.2
In Filters	51.7 ± 18.8	47.8 ± 2.7		46.7 ± 2.9	47.9 ± 2.4	48.0 ± 9.5		47.3 ± 0.7		46.3 ± 8.6		44.9 ± 12.5		28.2 ± 7.4	51.3 ± 30.6		42.9 ± 15.8		42.4 ± 5.1
*Leaves of Red Rooibos*																			
In Pods	27.6 ± 11.9	29.0 ± 2.6		29.9 ± 3.4	29.6 ± 0.4	34.8 ± 10.0		34.5 ± 13.8		16.2 ± 3.1		18.6 ± 2.7		17.9 ± 1.1	13.8 ± 5.8		28.9 ± 12.2		28.6 ± 14.4
In Filters	82.1 ± 22.1	70.2 ± 5.8		73.0 ± 5.9	78.6 ± 0.6	70.3 ± 25.2		69.9 ± 19.3		60.5 ± 10.9		73.4 ± 14.3		23.3 ± 2.5	82.6 ± 46.6		60.4 ± 9.4		75.3 ± 9.3
Average Value for Pods	37.5 ± 19.4	40.0 ± 17.3		37.4 ± 12.1	35.3 ± 12.7	22.8 ± 7.4		45.3 ± 21.3		30.2 ± 12.6		33.4 ± 17.1		32.1 ± 27.3	39.1 ± 29.9		36.2 ± 20.3		32.1 ± 20.8
Average Value for Filters	69.2 ± 24.1 *I*	61.5 ± 12.0 *I*		58.4 ± 11.7 *I*	64.4 ± 14.8 *I*	62.1 ± 19.5 *I*		62.2 ± 15.9 *I*		54.5 ± 9.8 *I*		59.1 ± 15.8 *I*		25.2 ± 6.0	66.2 ± 28.3 *I*		57.4 ± 21.1 *I*		59.7 ± 20.6 *I*
**Herbal Materials**	**Al**		**B**		**Ba**		**Ni**		**Li**		**As**		**Pb**			**Cd**		**Hg**	
*Flowers of Chamomile*																			
In Pods	43.2 ± 11.5		63.2 ± 24.2		75.5 ± 24.2		44.7 ± 15.9		9.0 ± 3.5		18.3 ± 12.7		46.7 ± 6.4			26.3 ± 13.3		n.c.	
In Filters	28.0 ± 5.6		42.0 ± 2.0		35.4 ± 6.2		54.5 ± 34.5		22.7 ± 1.3		37.3 ± 16.7		24.2 ± 16.2			11.1 ± 0.8		n.c.	
*Seeds of Fennel*																			
In Pods	24.8 ± 5.2		20.1 ± 3.9		30.3 ± 14.2		15.5 ± 8.0		0		4.3 ± 3.1		10.4 ± 5.3			0		n.c.	
In Filters	20.1 ± 2.7		39.7 ± 1.8		40.9 ± 27.4		37.3 ± 25.4		26.8 ± 10.8		26.2 ± 6.0		12.4 ± 6.1			0		n.c.	
*Roots of Licorice*																			
In Pods	28.6 ± 7.7		28.0 ± 6.4		21.4 ± 3.4		17.1 ± 6.6		n.c.		9.9 ± 3.3		16.5 ± 5.3			32.7 ± 1.7		n.c.	
In Filters	12.6 ± 3.6		47.2 ± 1.0		42.0 ± 5.3		43.8 ± 20.3		n.c.		42.7 ± 28.7		21.2 ± 15.0			0		n.c.	
*Leaves of Mate*																			
In Pods	24.6 ± 7.2		20.3 ± 3.2		24.6 ± 1.9		18.3 ± 2.4		6.9 ± 1.7		8.4 ± 4.1		16.8 ± 8.6			20.7 ± 1.2		0	
In Filters	12.7 ± 3.1		43.0 ± 1.3		37.6 ± 0.7		42.0 ± 8.0		31.4 ± 14.6		33.3 ± 14.8		15.2 ± 9.9			13.9 ± 3.2		0	
*Leaves of Mint*																			
In Pods	26.6 ± 5.5		22.4 ± 1.4		38.7 ± 5.1		24.3 ± 5.9		n.c.		10.9 ± 0.9		34.9 ± 15.0			35.7 ± 14.7		0	
In Filters	40.8 ± 7.4		40.1 ± 5.0		41.7 ± 4.6		75.9 ± 10.9		n.c.		37.9 ± 4.2		0			0		0	
*Leaves of Moringa*																			
In Pods	42.1 ± 5.6		50.7 ± 14.5		79.7 ± 15.5		38.7 ± 15.0		24.8 ± 4.7		34.9 ± 27.9		50.5 ± 31.7			86.4 ± 45.0		0	
In Filters	34.7 ± 2.3		30.0 ± 1.2		32.4 ± 2.8		32.3 ± 15.9		23.5 ± 8.2		38.2 ± 15.5		12.8 ± 2.9			0		0	
*Leaves of Red Rooibos*																			
In Pods	13.6 ± 2.0		37.0 ± 4.8		27.1 ± 2.8		32.8 ± 4.9		0		12.0 ± 3.3		28.3 ± 1.9			4.4 ± 1.2		n.c.	
In Filters	20.0 ± 1.3		37.2 ± 1.7		54.9 ± 35.1		41.8 ± 19.8		21.8 ± 2.8		60.9 ± 32.2		33.8 ± 21.6			9.2 ± 1.8		n.c.	
Average Value for Pods	29.1 ± 11.5		34.5 ± 18.5		42.5 ± 25.6		27.4 ± 13.6		8.1 ± 9.7 (n = 15)		14.1 ± 13.8		29.1 ± 18.9 *I*			29.5 ± 31.2 *I*		0 (n = 9)	
Average Value for Filters	24.1 ± 10.8		39.9 ± 5.5 *I*		40.7 ± 16.0		46.8 ± 22.3 *I*		25.2 ± 8.4 (n = 15) *I*		39.5 ± 19.2 *I*		17.1 ± 14.6			4.9 ± 6.0		0 (n = 9)	

Transfer rate (%) = (mg in herbal infusion/mg in herbal material) × 100; n.c., not computable (since the content of the element in the plant material is less than the LOQ, no value can be calculated); *I* indicates statistically higher results (*p* < 0.05) obtained by Mann Whitney U test.

**Table 5:** TPCs (mg) and percentages of the reference values (%) of mineral elements from the consumption of one cup (0.25 L) of herbal infusion.

Samples	TPC	K	Mg	Ca	Na	Fe	Mn	Zn	Cu	Cr	Mo	Co	Se	Al	B	Ba	Ni	Li	As	Pb	Cd
Reference values		RDA: 2000 mg/day	RDA: 375 mg/day	RDA: 800 mg/day	AI: 2000 mg/day	RDA: 14 mg/day	RDA: 2 mg/day	RDA: 10 mg/day	RDA: 1 mg/day	RDA: 40 µg/day	RDA: 50 µg/day	RDA: 20 µg/day	RDA: 55 µg/day	TWI: 1 mg/kg_bw_/week	UL: 10 mg/day	TDI: 200 µg/kg_bw_/day	TDI: 13 µg/kg_bw_/day	RDA: 1 mg/day	BMDL01: 0.3 µg/kg_bw_/day	BMDL01: 1.50 µg/kg_bw_/day	TWI: 2.5 µg/kg_bw_/week
Pods																					
Chamomile	46.3	0.32	0.70	0.15	<0.01	0.28	3.81	0.13	0.49	2.25	1.11	0.28	0.17	1.12	0.23	0.01	0.05	<0.01	0.09	0.06	0.07
Fennel	41.5	0.55	0.95	0.51	0.03	0.63	2.14	0.14	0.66	0.31	0.39	0.44	0.28	1.16	0.10	0.01	0.07	-	0.03	0.01	-
Licorice	120.5	0.28	0.72	0.24	0.01	0.36	10.08	0.06	0.20	0.73	0.18	0.76	0.06	0.45	0.12	0.09	0.03	-	0.09	0.01	0.13
Mate	207.1	0.42	0.83	0.35	<0.01	0.04	26.28	0.20	0.36	1.54	0.04	1.58	0.03	0.39	0.31	0.11	0.21	<0.01	0.08	0.01	0.74
Mint	133.3	0.77	1.50	1.10	0.01	3.40	4.05	0.38	1.14	0.82	1.38	1.12	0.10	1.23	0.02	0.07	0.07	-	0.12	0.06	0.14
Moringa	60.6	0.19	2.45	0.39	0.01	1.04	7.02	0.07	0.39	1.47	0.47	0.78	0.06	2.87	0.20	0.13	0.03	<0.01	0.15	0.02	0.07
Red rooibos	92.7	0.08	0.34	0.12	0.10	0.04	0.76	0.03	0.06	0.45	0.09	0.62	0.18	0.02	0.35	0.03	0.08	-	0.14	0.03	0.02
Filters																					
Chamomile	55.2	0.85	1.73	0.38	0.01	1.74	6.10	0.27	0.80	1.84	2.99	1.49	0.56	1.29	0.25	0.02	0.12	<0.01	0.25	0.02	0.16
Fennel	47.1	1.69	2.49	1.01	0.07	1.65	4.79	0.29	1.17	0.37	0.85	0.82	0.47	0.91	0.18	0.01	0.09	<0.01	0.11	0.02	-
Licorice	46.6	0.88	1.77	0.42	0.03	1.09	18.27	0.22	0.57	0.97	0.56	3.06	0.27	0.18	0.25	0.14	0.09	-	0.17	0.02	-
Mate	429.0	1.16	2.41	0.62	0.01	0.12	69.27	0.55	1.46	1.43	0.07	2.23	0.31	0.25	0.77	0.29	0.42	<0.01	0.46	0.01	0.27
Mint	203.7	1.44	3.03	2.25	0.02	9.59	9.73	0.41	1.57	1.57	3.18	1.98	0.21	1.49	0.03	0.06	0.21	-	0.28	0.00	-
Moringa	299.4	0.49	4.71	0.86	0.02	5.99	14.66	0.19	0.58	2.27	0.64	0.65	0.17	7.19	0.31	0.13	0.09	<0.01	0.61	0.01	-
Red rooibos	259.3	0.29	0.95	0.36	0.21	0.11	2.07	0.11	0.34	0.66	0.62	1.26	0.40	0.03	0.37	0.04	0.07	<0.01	0.75	0.06	0.04

Abbreviations: TPC, total polyphenol content; AI, Adequate Intake; RDA, Recommended Dietary Allowance; TDI, Tolerable Daily Intake; TWI, Tolerable Weekly Intake; UL, Tolerable Upper Intake Level; BMDL01, Benchmark Dose Lower Confidence Limit 01.

## Data Availability

The original contributions presented in the study are included in the article/Supplementary Materials; further inquiries can be directed to the corresponding author.

## Supplementary Materials

The following supporting information can be downloaded at: https://www.mdpi.com/article/10.3390/foods13132145/s1, Table S1. Instrument operating conditions for inductively coupled plasma mass spectrometry (ICP-MS) analyses; Table S2. Method performance parameters for the investigated elements.
