# Supplementary material for: Total Polyphenol Contents and Mineral Profiles in Commercial Wellness Herbal Infusions: Evaluation of the Differences between Two Preparation Methods
Source: Foods. 2024 Jul 5;13(13):2145. doi: 10.3390/foods13132145 (PMC11241193; doi:10.3390/foods13132145)
Supplement: Supplementary file 1 [file foods-13-02145-s001.zip › foods-3046759-supplementary.pdf]

Total polyphenols content and minerals profile in commercial wellness herbal infusions: evaluation of the differences between two preparation methods.

Vincenzo Lo Turco, Vincenzo Nava, Angela Giorgia Potortì *, Benedetta Sgro, Maria Aurora Arrigo and Giuseppa Di Bella

Department of Biomedical and Dental Sciences and of Morphological and Functional Images (BIOMORF), University of Messina, V.le G. Palatucci, 98168, Messina, Italy.

* Correspondence: agpotorti@unime.it (A.G.P.)

Table S1. Instrument operating conditions for inductively coupled plasma mass spectrometry (ICP-MS) analyses.

Nebulizer	Concentric PFA
RF power	1550 W
Sample depth	5 mm
Sample introduction flow rate	0.93 mL/min
Interface pressure	1.89 Pa
Plasma/auxiliary/argon carrier gas flow rate	14 L/min, 0.8 L/min and 1.1 L/min
Helium collision gas flow rate	4.7 mL/min
Dwell time	1s
Vacuum	$< 7.5\ 10^{-7}$ Pa
Extract lens 1 voltage	1.5 V
Spray chamber temperature	2.7 °C
Nebulizer pump	0.1 Hz
integration times were	0.5 s/point for Fe, Se and As<br>0.1 s/point for the other elements
Monitored isotopes	^{7}Li, ^{11}B, ^{23}Na, ^{24}Mg, ^{27}Al, ^{39}K, ^{40}Ca, ^{52}Cr, ^{55}Mn, ^{56}Fe, ^{59}Co, ^{60}Ni, ^{63}Cu, ^{66}Zn, ^{75}As, ^{80}Se, ^{98}Mo, ^{111}Cd, ^{138}Ba, and ^{208}Pb

Table S2. Method performance parameters for the investigated elements.

Elements	R^2	LOD (µg/Kg)	LOQ (µg/Kg)	NIST1570A			Herbal infusion		
				Accuracy (%)	Repeatability (RSD%)	Intermediate precision (RSD%)	Recovery** (%)	Repeatability** (RSD%)	Intermediate precision (RSD%)**
K	0.9981	0.067	0.221	92.73	3.73	4.89	93.18	3.05	4.82
Mg*	0.9996	0.119	0.036	96.42	2.17	3.32	95.27	2.94	3.67
Ca	0.9976	0.530	1.750	91.97	3.41	4.75	93.62	4.04	6.73
Na	0.9985	0.595	1.962	94.98	4.93	6.01	96.04	3.65	4.65
Fe*	0.9998	0.004	0.014	96.00	3.22	4.28	97.59	2.99	3.86
Mn	0.9998	0.002	0.007	99.61	2.74	3.80	101.33	3.17	4.53
Zn	0.9994	0.017	0.056	96.47	1.84	3.05	93.89	1.45	2.81
Cu	0.9996	0.003	0.010	98.45	2.18	3.86	96.34	3.49	4.17
Cr*	0.9993	0.002	0.005	98.50	4.09	5.43	98.93	4.27	6.15
Mo*	0.9998	0.001	0.002	100.50	2.31	3.57	97.12	2.43	4.11
Co	0.9997	0.001	0.002	98.73	3.12	5.63	95.53	2.82	3.49
Se	0.9996	0.020	0.062	98.96	1.45	2.97	96.21	2.05	3.26
Al	0.9994	0.024	0.080	97.76	2.37	3.86	100.64	1.99	2.83
B*	0.9994	0.003	0.010	94.96	3.18	4.60	96.25	5.17	6.79
Ba*	0.9995	0.001	0.002	97.50	3.66	4.82	98.79	3.44	5.03
Ni	0.9998	0.003	0.010	99.67	3.34	4.55	102.19	3.55	4.87
Li*	0.9997	0.003	0.010	96.00	2.87	4.07	94.73	2.02	3.47
As	0.9998	0.001	0.003	97.06	1.48	2.94	99.38	1.56	3.09
Pb*	0.9999	0.001	0.003	102.50	5.11	6.89	98.17	4.46	5.92
Cd	0.9999	0.001	0.003	100.21	3.86	5.05	99.56	3.35	4.90
Hg	0.9998	0.001	0.003	97.31	4.15	5.72	99.98	3.82	5.06

R^2, determination coefficient; LOD, limits of detection = 3.3σ/S; LOQ, limits of quantification = 10σ/S; RSD, relative standard deviations. * Elements not included in to certified matrix and added at 1 ppm concentration. ** Parameters calculated by adding know amounts of elements in a sample previously analysed.
